# Sex-Specific Association of Ambient Temperature With Urine Biomarkers in Southwest Coastal Bangladesh

**DOI:** 10.1016/j.ekir.2024.03.002

**Published:** 2024-03-11

**Authors:** Hoimonty Mazumder, Momenul Haque Mondol, Mahbubur Rahman, Rizwana Khan, Solaiman Doza, Leanne Unicomb, Farjana Jahan, Ayesha Mukhopadhyay, Konstantinos C. Makris, Alberto Caban-Martinez, Romaina Iqbal, Faruk Ahmed, Lota Creencia, Mohammad Shamsudduha, Fawaz Mzayek, Chunrong Jia, Hongmei Zhang, Anwar Musah, Lora E. Fleming, Xichen Mou, Csaba P. Kovesdy, Matthew O. Gribble, Abu Mohd Naser

**Affiliations:** 1 Division of Epidemiology, Biostatistics, and Environmental Health; School of Public Health, The University of Memphis, Memphis, Tennessee, USA; 2 School of Population and Public Health, University of British Columbia, Vancouver, British Columbia, Canada; Department of Statistics, University of Barishal, Barishal-8254, Bangladesh; 3 International Centre for Diarrheal Disease Research, Bangladesh, Bangladesh; 4 Environmental and Occupational Health, School of Biological and Population Health Sciences, Oregon State University, Oregon, USA; 5 Cyprus International Institute for Environmental and Public Health, School of Health Sciences, Cyprus University of Technology, Limassol, Cyprus; 6 Department of Public Health Sciences, University of Miami Miller School of Medicine, Miami, Florida, USA; 7 Department of Community Health Sciences, Aga Khan University, Pakistan; 8 Department of Engineering Technology, The University of Memphis, Memphis, Tennessee, USA; 9 College of Fisheries and Aquatic Sciences, Western Philippines University, Palawan, Philippines; 10 Institute for Risk and Disaster Reduction, University College London, London, UK; 11 Department of Geography, University College London, London, UK; 12 European Centre for Environment and Human Health, University of Exeter Medical School, Truro, Cornwall, UK; 13 Division of Nephrology, University of Tennessee Health Science Centre, Memphis, Tenessee; USA; 14 Department of Medicine, Division of Occupational, Environmental, and Climate Medicine, University of California, San Francisco, San Francisco, California, USA

**Keywords:** climate and health, environment, environmental determinants of health, GeoHealth, planetary health, renal elimination

## Abstract

**Introduction:**

Men are vulnerable to ambient heat-related kidney disease burden; however, limited evidence exists on how vulnerable women are when exposed to high ambient heat. We evaluated the sex-specific association between ambient temperature and urine electrolytes, and 24-hour urine total protein, and volume.

**Methods:**

We pooled a longitudinal 5624 person-visits data of 1175 participants' concentration and 24-hour excretion of urine electrolytes and other biomarkers (24-hour urine total protein and volume) from southwest coastal Bangladesh (Khulna, Satkhira, and Mongla districts) during November 2016 to April 2017. We then spatiotemporally linked ambient temperature data from local weather stations to participants' health outcomes. For evaluating the relationships between average ambient temperature and urine electrolytes and other biomarkers, we plotted confounder-adjusted restricted cubic spline (RCS) plots using participant-level, household-level, and community-level random intercepts. We then used piece-wise linear mixed-effects models for different ambient temperature segments determined by inflection points in RCS plots and reported the maximum likelihood estimates and cluster robust standard errors. By applying interaction terms for sex and ambient temperature, we determined the overall significance using the Wald test. Bonferroni correction was used for multiple comparisons.

**Results:**

The RCS plots demonstrated nonlinear associations between ambient heat and urine biomarkers for males and females. Piecewise linear mixed-effects models suggested that sex did not modify the relationship of ambient temperature with any of the urine parameters after Bonferroni correction (*P* < 0.004).

**Conclusion:**

Our findings suggest that women are as susceptible to the effects of high ambient temperature exposure as men.

## Graphical abstract

**Figure:**
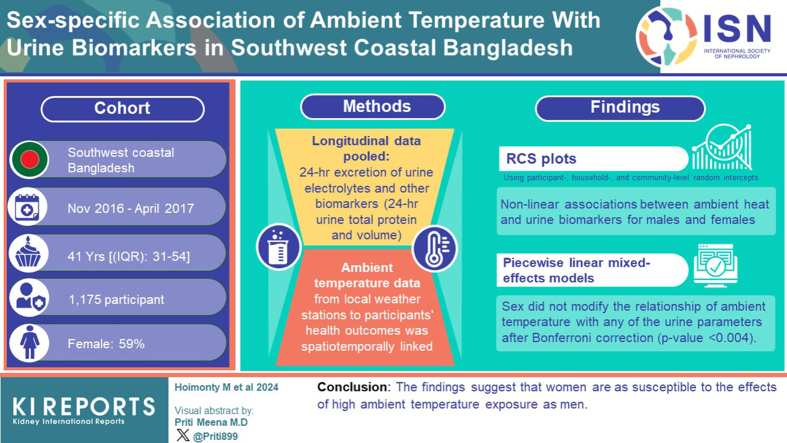

Millions of people worldwide experienced higher ambient temperatures and were exposed to heatwaves during the summer of 2023. July 2023 was recorded as the world’s hottest month ever since 1980, representing the fast-paced effect of climate change.^1^ Extreme heat exposure is linked to heat stress,^2^ heatstroke,^3^ kidney injury^4^ and exacerbation of congestive heart failure.^5^ Studies have identified a higher risk of nephropathies among outdoor agricultural workers attributed partly to high ambient temperatures in Central American and South Asian countries.^6^, ^7^, ^8^ Studies also suggest high ambient heat is associated with a higher prevalence of calcium-containing kidney stones in tropical regions.^9^^,^^10^

The kidney plays a pivotal role in human physiology that allows the filtering of metabolic waste, reabsorption of vital nutrients, body fluid and temperature regulation, and electrolytes and acid-base balances. Urine biomarkers (e.g., electrolytes and total protein) and volume can be indicative of renal structural and regulatory function and the body’s overall hydration status. In exposure to high ambient heat, fluid and electrolytes depletion caused by profuse sweating may cause hypohydration. Chronic or repeated dehydration or hypohydration are found to be associated with an increased risk of nephropathies, including nephrolithiasis especially during heat exposure.^11^, ^12^, ^13^, ^14^ Therefore, low urine volume and concentrated urine are the clinical signs manifesting hypohydration, which may increase the risk of developing nephropathies and nephrolithiasis.

Many coastal communities are increasingly experiencing dual burden of climate changes, namely high ambient temperature and higher drinking water salinity due to saltwater intrusion. For example, residents of tropical coastal Bangladesh have been suffering from potable water scarcity due to seasonal variability in freshwater supply exacerbated by saltwater intrusion.^15^^,^^16^ Therefore, coastal populations of Bangladesh may experience chronic hypohydration due to limited options of potable water sources to rehydrate themselves, making them more vulnerable to kidney diseases when high ambient heat causes increased water loss due to sweating.

Ambient temperature-induced pathophysiologic responses are also contingent on many sociobehavioral and cultural attributes. Poverty, unhealthy housing structure, the nature of work (e.g., manual agricultural labor, farming work, and fishing), and precarious working environment may lead to prolonged and intensive atmospheric heat exposure with less adaptive capacities;^17^ thus increasing the risk of adverse kidney outcomes and other health hazards.

Epidemiological studies have reported that men are particularly at high risk of developing nephropathies and nephrolithiasis globally.^18^^,^^19^ Because men largely involve in outdoor work by exposing themselves directly under the sun, prolonged exposure to high ambient heat is very likely. However, women are also involved in strenuous outdoor activities in the vicinity of their homes and fields^20^ in many cultures, making them similarly exposed to ambient heat.

Sex could be a biological variable that influences dimorphic physiological responses against ambient heat exposure, making men more vulnerable. Men typically have lower urine volume than women during high ambient temperatures,^19^ indicating that sex may affect thermoregulation; however, this does not mean that women are less vulnerable, if the heat exposure is similar. Therefore, the role of sex, to some extent, has been contentious, especially when related to strenuous work in atmospheric heat, reportedly decreasing kidney function in both men and women.^21^

Unlike men, the health impact of chronic exposure to ambient heat on women is inconclusive. Disaggregated data by sex and sex-stratified analyses considering similar heat exposure across population may inform vulnerability and guide clinical and public health interventions to protect all groups affected from adverse health consequences of raised ambient temperature.

In this study, we explored whether men and women have similar concentration and excretion of renal biomarkers (e.g., urine electrolytes [sodium, potassium, chloride, calcium, and magnesium], 24-hour excretion of urine total protein, and volume) when exposed to a range of ambient temperatures spanning through the winter and summer months in a tropical coastal region of Bangladesh.

## Methods

### Study Setting and Design

We used urine electrolytes (sodium, potassium, chloride, calcium, and magnesium), 24-hour total protein, and volume data from a stepped-wedge randomized controlled trial conducted in 3 southwest coastal districts (Khulna, Satkhira, and Bagerhat) of Bangladesh (Supplementary Figure S1) from November 2016 to April 2017,^22^^,^^23^ covering both dry winter and early dry summer. The trial followed 1175 participants from 542 households. We collected urine samples during 5 visits to evaluate the health effects of a low salinity drinking water intervention provided by managed aquifer recharge.

Managed aquifer recharge systems infiltrate fresh rainwater and pondwater into the aquifer system to create a buffer of freshwater in the aquifer and provide a year-round supply of drinking water. The salinity of managed aquifer recharge water is lower compared to the brackish groundwater.^23^ All participants gained access to the intervention managed aquifer recharge water supply at some point in the study period during the second to fifth visits. Urine samples were collected from both intervention and control phases in all 5 consecutive months during the study period, making a total of 5624 person visits. Details of the study design, selection and enrollment of participants have been reported elsewhere.^22^ Each individual participant’s visit data were linked to temperature data of the visit day.

### Ambient Temperature Data

**Figure 1:**
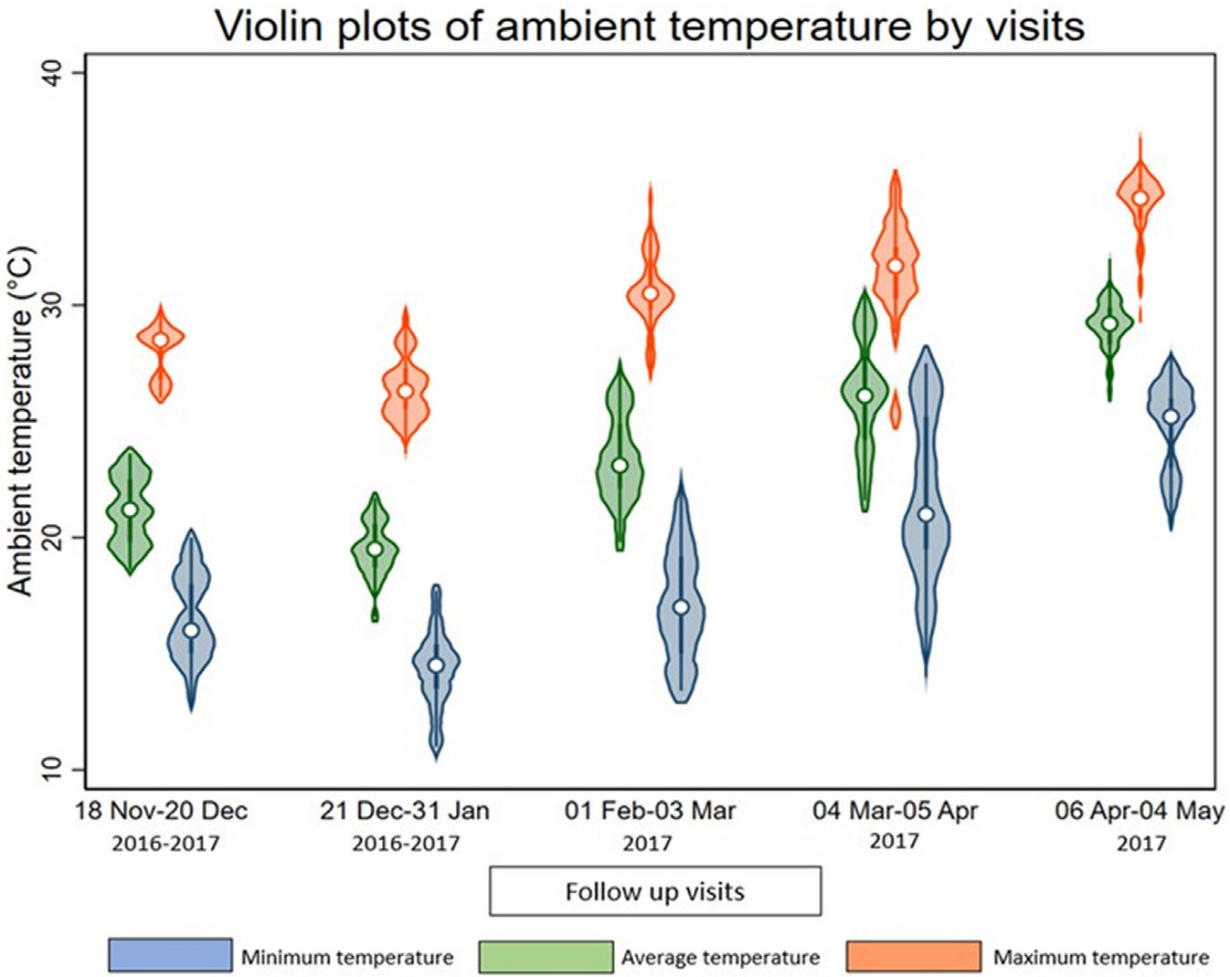
Distribution of ambient temperature (minimum, average, and maximum) during the study period (December 2016–April 2017).

The daily ambient temperature data were collected from the Bangladesh Meteorological Department. We collected daily ambient average, maximum, and minimum temperature data for the study period from 3 local weather stations situated in the study areas (Khulna, Satkhira, and Bagerhat districts). The distribution of average, maximum and minimum ambient temperature was illustrated in Figure 1. We calculated 3 linear distances for each participant residence, considering the geolocation of the residence and each weather station (Khulna, Mongla, and Satkhira) along the surface of a mathematical model of the earth.^24^ We then assigned temperature data to each participant from the closest weather station to their residence that provided the shortest linear distance.

The surface elevation of Bangladesh is below 10 meters with respect to mean sea level,^25^ and the spatial variability of ambient temperature of the region for any given time is low.^26^ The mean temperature in Khulna region during winter (December–February) is 20.53 °C, which increases to 28.65 °C during the summer (March–May).^26^ Notably, all 3 districts of this study are located in the Khulna region. April is the warmest month in Bangladesh, with an annual mean temperature of 28.06 °C, whereas January is the coldest month when the average temperature across the country varies between 12 and 18 °C.^26^^,^^27^

### Urine Sample Collection and Processing

During each monthly visit, 24-hour urine samples were collected from the study participants.^22^ A total of 5 visits were scheduled 4-weeks apart, each for collecting samples during winter and summer time. For collecting urine samples, each participant received a 4-liter container. Study participants were instructed to specify the collection period from the second morning void to the next morning’s first void. Participants were provided with a small pot to transfer all voids into the 4-liter container.^28^ Aliquots of each participant’s urine sample were maintained for measuring urine volume, electrolytes, and total protein.^22^ The field research assistants documented the 24-hour urine volume, obtained 25 ml of urine samples after stirring, and then transported them to a field laboratory at 2 to 8 °C within 6 hours of collection.

In the field laboratory, the direct ion-selective method was used to measure urinary sodium and potassium with a semi-auto electrolyte analyzer (Biolyte2000, Bio-care Corporation, Taiwan, coefficient of variation: ± 5%). The photometric titration method with a semi-auto biochemistry analyzer was used to assess urine calcium and magnesium, and the colorimetric method for urine protein, using a semiauto analyzer (Evolution 3000, BSI, coefficient of variation: <1%). The concentration of urine volume and biomarkers (electrolytes and total protein) were expressed in measurement units such as mmol/l or mg/l, as appropriate. Finally, 24-hour urine excretions of electrolytes and total protein were calculated by multiplying their concentrations by the participant 24-hour urine volume.

### Confounders and Covariates Data

We collected data on several potential confounders, including demographic and individual information of study participants (e.g., age, weight, height, smoking status, alcohol consumption, physical activities, religion, and hours of self-reported sleep duration) during the 5 consecutive visits; height was measured only once. These confounders were selected based on their potential influences on ambient temperature exposure and chronic diseases, including kidney function.

Anthropometric measurements included body mass index, calculated using the participants’ weight and height. Sociodemographic factors included age, religion, and household wealth quintiles.^29^ Participant age influences kidney function; age also determines the ambient temperature exposure because middle-aged participants are more likely to work outside compared to the old participants. Obesity may result in a lower exposure to ambient heat due to decreased physical activity. Household wealth was estimated by collective information for each household on ownership of refrigerator, television, mobile phone, motorcycle, bicycle, sewing machine, chairs, table, wristwatch, wardrobe, wooden cot, motor pump, rice husking machine, motorized rickshaw, car, and access to electricity. The household wealth score was estimated by a principal component analysis using ownership of household assets, thereafter, categorized into wealth quintiles.^30^

Given that religion strongly influences the dietary pattern and clothing of the participants, we considered religion a covariate. Muslims in this region typically consume red meat, whereas Hindus are mostly vegetarian.^31^ Behavioral factors included smoking status (never, current, former),^32^ physical exercise,^33^ alcohol use,^34^ and duration of sleep.^35^

We used the WHO STEPS questionnaire to measure participants' physical activity and categorization into sedentary, moderate, and vigorous.^36^ This questionnaire covers physical activity performed in 3 domains: occupational physical activity, transport-related physical activity, and physical activity during discretionary or leisure time.^37^ Water salinity was considered a potential covariate because it is associated with both ambient temperature and urinary electrolyte concentration.^38^^,^^39^ Because humidity affects both ambient temperature and kidney diseases,^40^ we also adjusted for humidity in the statistical models.

### Statistical Analyses

We investigated the distributions of outcome variables (e.g., urine electrolytes, total protein, and urine volume) (Supplementary Figure S2) and did natural logarithm transformation of the skewed biomarkers (e.g., concentration of sodium, potassium, chloride, calcium, magnesium, and 24-hour excretion of calcium, magnesium, and total protein). For the skewed urine electrolytes and protein, few values were outside of the plausible biological range. We performed winsorisation^41^ and replaced those values with percentile values (e.g., 0.2 and 99.8th percentiles).

We created violin plots illustrating the distribution of urine electrolytes, and 24-hour total protein across different tertiles of ambient temperatures for males and females. We also illustrated how the distribution of 24-hour urine volume varies across different ambient temperatures with respect to age and sex categories. All outcome variables and covariates such as age, body mass index, concentration and 24-hour excretion of urine creatinine, water salinity, and humidity were continuous, whereas other covariates such as religion, smoking, alcohol use, physical exercise, sleep duration, and household wealth quintile were categorical.

To visually illustrate nonlinearity of sex-stratified relationships between daily ambient temperature and urine electrolytes, 24-hour urine protein and volume, we created RCS plots by employing linear mixed-effects models. We assumed 3 levels of clustering (individual, household, and community) and applied random intercepts approach to account for the clustering at different levels. For creating the RCS plots, 4 knots were positioned at the 5th , 35th , 65th , and 95th percentile distributions of daily ambient temperature according to Harrell’s rule.^42^ The expected conditional mean outcome for the *i*th participant is modeled in Equation 1, where *i*th participant in the *j*th household of *k*th community measured at *t*th visit; ${Temp}_{t}$ is the temperature at *t*th visit, followed by *p* covariates such as age and body mass index (depending on settings of model 1–4); $u_{jk}$ and $v_{k}$ denote the random intercepts to consider individual, household, and community clustering. The betas are the coefficients of corresponding exposures.

Equation:

$${E\;[Y}_{ijkt}]=\beta_{0}+\beta_{1}{Temp}_{t}+\beta_{2}{Age}_{ijk}+\ldots +\beta_{p+1}{BMI}_{ijk}+u_{jk}+v_{k}+\varphi_{ijk};\;i=1,2,\ldots .,1175;j=1,2,\ldots \ldots ,542;and\;k=1,2,\ldots ..x$$

Upon visualizing nonlinear relationships of ambient temperature with urine electrolytes and other biomarkers (24-hour urine total protein and volume), we ran piece-wise linear mixed-effect models using similar random intercepts approach for accounting the level cluster effects (individual, household, and community) to demonstrate the ambient-temperature-urine-biomarker relationships for different segments of ambient temperature distributions. We defined ambient temperature segments based on the inflection points of the RCS plots described above, and reported cluster-adjusted standard errors for inference.^43^^,^^44^ We examined sex as an effect modifier in assessing temperature-dependent changes in urinary biomarkers for each of the ambient temperature segment and reported the *P*-value of the overall significance of the interaction terms from all segments using the Wald test.^45^

We implemented separate models for average, minimum, and maximum daily ambient temperature. We sequentially adjusted for covariates in piece-wise models: model 1 evaluated the unadjusted association between exposure (ambient temperature) and outcomes (urine electrolytes, total protein, and volume). In model 2, individual risk factors such as age, body mass index, and creatinine were adjusted. Because creatinine-adjustment is a common method for adjusting dilution of urine samples, we adjusted for urinary creatinine measurements in this model.^46^^,^^47^ Model 3 was additionally adjusted for religion, physical exercise, smoking status, sleep duration, alcohol consumption, and household wealth; and model 4 further accounted for the time of the visit, participants’ drinking water salinity, and humidity. The time of the visit was used as a proxy for time-varying confounders.

We back-transformed the coefficients of log-transformed variables and reported geometric mean ratios. Because we have 12 outcomes, we used Bonferroni correction to account for the multiple comparisons (e.g., urine volume and different biomarkers) in the final model (model 4), adjusting the alpha-level at 0.004 (0.05/12) for (α = 0.05/12 or 0.004) multiple comparisons. We used STATA SE-17.0 for statistical analyses.^48^

We also ran piece-wise linear quantile mixed models with participant-level random intercept to models 1 to 4 described above, where we evaluated the relationships between average daily ambient temperature and medians of urine electrolytes, total protein, and volume. The similar specifications described above for piece-wise linear mixed effects were used for the quantile models.

### Sensitivity Analyses

We conducted several sensitivity analyses. We did a stratified analyses for participants who did not self- report any comorbidities (e.g., heart diseases, kidney disease, diabetes, and stroke). Because we collected 24-hour urine of the study participants in a rural nonclinical setting, there may be undercollection or overcollection of 24-hour urine samples. We used a creatinine-based measure of completeness assessment of 24-hour urine sample collection and implemented a stratified analyses among participants with complete 24-hour urine collection based on creatinine index >0.7.^28^^,^^49^

We created RCS plots using linear mixed-effect model for urine electrolytes and urine total protein after excluding their outliers. Furthermore, stratified analyses were performed to create RCS plots using linear mixed-effects models for women aged ≤49 years and those aged ≥49 years. This aimed at investigating potential variations in the impact of average ambient temperature on urine biomarkers for reproductive and postmenopausal women, which could imply the thermogenic effects of female sex hormones on outcomes of interest.^50^^,^^51^ We chose the age cutoff of 49 years based on the existing literature from Bangladesh, which suggest that women aged 49 or younger are usually considered to be women of reproductive age.^52^, ^53^, ^54^

### Ethical Considerations

The protocol of this study was reviewed and approved by ethical review committee (PR-15096) of International Center for Diarrheal Disease Research, Bangladesh. Informed consents were obtained from all the study participants, including household heads at the beginning of the study. Participant’s privacy and confidentiality were maintained throughout data storage, analysis, and dissemination phase; collected data were stored anonymized without any personal identifier. University of Memphis institutional review board identifies this article as nonhuman subject research because we did not have direct contact with participants and we analyzed deidentified data as secondary analysis.

## Results

The median age of the sample population was 41 years (interquartile range: 31–54). The majority were women (59%), married (96%), and belonged to the Hindu religion (58 %). Fifty-one percent of study participants were never smokers, and 97% did not drink alcohol. In rural Bangladesh, men are the breadwinners and usually work outside, whereas women stay at home and manage household chores. This explains higher proportion of female participants in our study.

An overview of the characteristics of participants, including sex-stratified distribution, is presented in Table 1. In the study area during the 5-month study period (December, 2016–April, 2017), the highest daily mean temperature was 30.4 °C in April; whereas in January, the lowest mean temperature was recorded (16.6 °C). Violin plots of urine electrolytes and 24-hour total protein excretion across different tertiles of ambient temperatures suggest similar mean distribution for men and women (Supplementary Figures S5–S9). We found the similar mean distribution of 24-hour urine volume in relation to ambient temperatures with respect to sex and age categories (Supplementary Figures S3 and S4).

**Table 1:** Sex-specific distribution of individual characteristics and urine biomarkers of the participants (*N* = 1175)

Characteristics	Overall (*N* = 1175)	Male (*n* = 477)	Female (*n* = 698)
Age, yr, mean (95% CI)	42.7 (41.8–43.6)	46.5 (45.1–47.8)	40.2 (39.2–41.2)
BMI, mean (95% CI)	22.2 (21.9–22.48)	21.7 (21.4–22.0)	22.6 (22.2–22.9)
Religion, % (*n*)			
Islam	41.9 (492)	37.9 (181)	44.6 (311)
Hindu	58.1 (683)	62.1 (296)	55.4 (387)
Smoking, % (*n*)			
Never	50.7 (596)	20.6 (98)	71.4 (498)
Former	9.1 (107)	22.0 (105)	0.3 (02)
Current	40.2 (472)	57.4 (274)	28.4 (198)
Alcohol use, % (*n*)			
Yes	2.89 (34)	6.71 (32)	0.29 (02)
No	97.1 (1141)	93.3 (445)	99.7 (696)
Physical exercise, % (*n*)			
Sedentary	40.4 (475)	29.1(139)	48.1 (336)
Moderate	31.2 (366)	31.7 (151)	30.8 (215)
Vigorous	28.4 (334)	39.2 (187)	21.1 (147)
Sleep, % (*n*)			
<6 h	21.1 (248)	21.2 (101)	21.0 (147)
6–9 h	66.8 (785)	67.3 (321)	66.5 (464)
≥ 9 h	12.1 (142)	11.5 (55)	12.5 (87)
Household wealth quintile, % (*n*)			
1st quintile	17.6 (207)	17.6 (84)	17.6 (123)
2nd quintile	18.4 (216)	16.4 (78)	19.8 (138)
3rd quintile	19.2 (225)	19.1 (91)	19.2 (134)
4th quintile	20.8 (244)	20.3 (97)	21.1 (147)
5th quintile	24.1 (283)	26.6 (127)	22.4 (156)
Urinary creatinine concentration in mmol/l, median (IQR)	10.9 (8.3–14.0)	12.8 (9.5–15.9)	10.0 (7.8–12.5)
24h urinary creatinine excretion in mmol/l, median (IQR)	21.2 (12.9–33.4)	25.1 (14.1–39.1)	19.3 (12.2–29.5)
Urinary biomarkers’ concentration, median (IQR)			
Sodium (mmol/l)	80.5 (55.9–115.3)	77.6 (53.5–113.9)	82 (57.3– 116.4)
Potassium (mmol/l)	16.4 (11.9–22.9)	16.4 (11.9–23.3)	16.3 (11.9–22.6)
Chloride (mmol/l)	86.3 (58.7–124.8)	83.5 (57–125.6)	87.9 (60.2–124.5)
Calcium (mmol/l)	1.8 (1.05–2.9)	1.8 (1.05–2.9)	1.8 (1.05–2.9)
Magnesium (mmol/l)	1.8 (1.1–2.7)	1.8 (1.1–2.8)	1.7 (1.1–2.6)
24h urinary excretion of urine biomarkers, median (IQR)			
Sodium (mmol/24h)	157.8 (118.6–207.4)	155.9 (115–203.9)	159.4 (120.4–209.7)
Potassium (mmol/24h)	32.5 (24.0–42.2)	32.9 (24.0–43.2)	32.2 (24.1–41.6)
Chloride (mmol/24h)	166.9 (126.8–222.4)	164.9 (124.4–221.6)	168.4 (128.3–223.2)
Calcium (mmol/24h)	3.4 (1.9–5.4)	3.4 (1.9–5.5)	3.4 (1.9–5.4)
Magnesium (mmol/24h)	3.3 (2.1–4.8)	3.4 (2.2–5.1)	3.3 (2.1–4.7)
Urine total protein (mg/24h)	207.7 (122.4–326.2)	201.5 (119.4–322.2)	210.9 (124.7–328.5)
Urine volume (liter/24h)	1.9 (1.4–2.6)	1.9 (1.3–2.7)	1.9 (1.4–2.6)

BMI, body mass index; CI, confidence interval; IQR, interquartile range.

**Figure 2:**
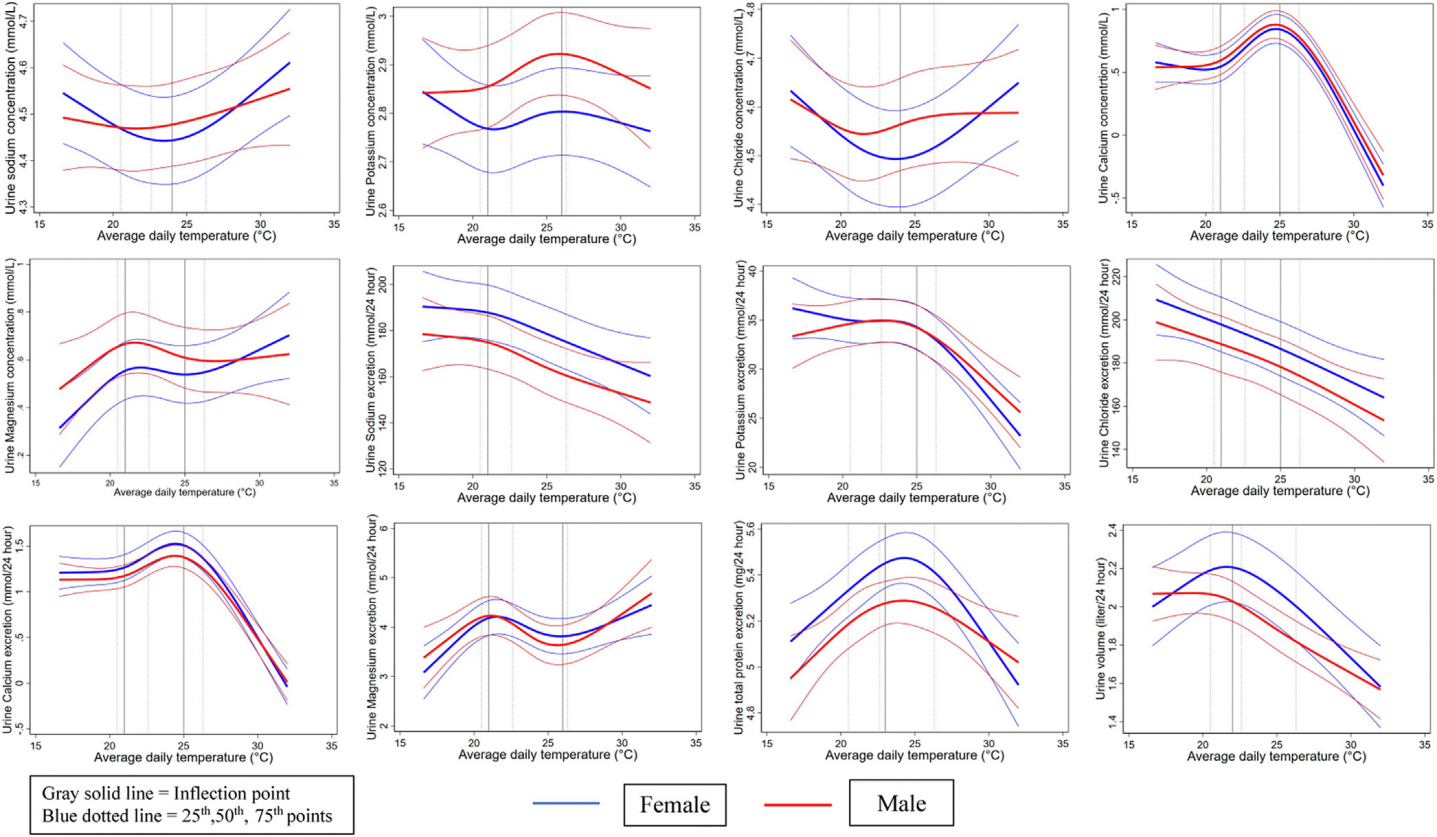
Restricted cubic spline plots with their 95% confidence bands using linear mixed-effect model demonstrating sex-stratified association between the concentration and 24-hour excretion of urinary electrolytes (sodium, potassium, chloride, calcium, and magnesium) and 24-hour excretion of urine total protein and volume with average ambient temperature, adjusted for age, body mass index, physical exercise, smoking, alcohol consumption, sleep duration, religion, household wealth, time of visit, drinking water salinity, and humidity.

The RCS plots illustrated a similar sex-specific relationship between daily average temperature with urine electrolyte concentrations (Figure 2). Both men and women had a similar U-shaped relationship of average temperature with urine sodium and chloride concentrations. Urine potassium concentration also exhibited a similar nonlinear relationship in both men and women. We found similar inverse associations between average temperature and 24-hour urinary excretion of sodium, potassium, chloride, total protein, and urine volume for both men and women. In addition, we observed similar positive relationships of the concentrations and 24-hour excretions of urine calcium and magnesium with high ambient heat. RCS plots reflected similar associations between minimum and maximum ambient temperature with urine electrolytes and other biomarkers (Supplementary Figures S10 and S11).

The Wald test for the interaction terms in piece-wise linear mixed models did not provide any evidence of modification of average ambient temperature and urine biomarkers' associations by sex (Tables 2 and 3). Likewise, we found similar associations between ambient temperatures (minimum and maximum) and urine electrolytes, total protein, and volume, where sex had no effect modification (Supplementary Tables S1–S4). From the piece-wise linear quantile models, we observed similar associations for average daily ambient temperature and median concentrations of urine electrolytes and other biomarkers for men and women (Supplementary Tables S5 and S6). Analyses conducted after excluding outliers revealed similar associations between average temperature and urine electrolytes and total protein (Supplementary Figure S12).

**Table 2:** Piecewise linear regression model showing associations between daily average temperature and concentrations of urine biomarkers for different average temperature segments using individual, household, and community level random intercepts

Temperature segments	Sex	Model 1	*P*-value	Model 2	*P*-value	Model 3	*P*-value	Model 4	*P*-value
Urine sodium concentration (mmol/l)									
<24 °C	F	1.00 (0.97, 1.04)	0.869	1.00 (0.96, 1.03)	0.750	1.00 (0.96, 1.03)	0.752	0.93 (0.98, 1.12)	0.630
	M	1.01 (0.97, 1.06)		1.01 (0.97, 1.05)		1.01 (0.97, 1.05)		0.95 (1.00, 1.12)	
≥24 °C	F	1.19 (1.15, 1.23)		1.20 (1.15, 1.25)		1.20 (1.16, 1.25)		1.13 (1.06, 1.19)	
	M	1.17 (1.12, 1.22)		1.17 (1.13, 1.22)		1.17 (1.13, 1.22)		1.09 (1.03, 1.16)	
Urine potassium concentration (mmol/l)									
<21 °C	F	0.77 (0.85, 1.01)	0.225	0.92 (0.84, 1.01)	0.243	0.92 (0.85, 1.01)	0.246	0.91 (0.83, 1.00)	0.176
	M	0.99 (0.89, 1.09)		0.99 (0.89, 1.09)		0.99 (0.89, 1.09)		0.99 (0.89, 1.09)	
≥21 to <26 °C	F	1.13 (1.07, 1.17)		1.12 (1.07, 1.17)		1.12 (1.07, 1.17)		1.03 (0.97, 1.09)	
	M	1.16 (1.09, 1.23)		1.15 (1.09, 1.22)		1.15 (1.09, 1.22)		1.05 (0.99, 1.13)	
≥26 °C	F	0.98 (0.92, 1.05)		1.00 (0.93, 1.07)		1.00 (0.93, 1.07)		1.12 (1.02, 1.21)	
	M	1.01 (0.94, 1.11)		1.03 (0.95, 1.12)		1.03 (0.95, 1.12)		1.15 (1.04, 1.27)	
Urine magnesium concentration (mmol/l)									
<21 °C	F	1.20 (0.95, 1.49)	0.173	1.20 (0.95, 1.49)	0.145	1.19 (0.94, 1.49)	0.161	1.15 (0.83, 1.60)	0.517
	M	0.99 (0.76, 1.27)		1.00 (0.76, 1.31)		1.00 (0.77, 1.31)		0.99 (0.67, 1.46)	
≥21 to <25 °C	F	1.26 (1.06, 1.48)		1.26 (1.05, 1.49)		1.26 (1.05, 1.49)		0.88 (0.70, 1.11)	
	M	1.36 (1.08, 1.72)		1.34 (1.06, 1.68)		1.34 (1.06, 1.68)		0.94 (0.68, 1.31)	
≥25 °C	F	1.43 (1.26, 1.62)		1.43 (1.26, 1.62)		1.43 (1.26, 1.62)		0.89 (0.70, 1.12)	
	M	1.43 (1.20, 1.72)		1.46 (1.21, 1.75)		1.46 (1.22, 1.75)		0.89 (0.66, 1.17)	
Urine calcium concentration (mmol/l)									
<21 °C	F	0.90 (0.64, 1.26)	0.872	0.90 (0.64, 1.26)	0.829	0.89 (0.64, 1.25)	0.820	1.02 (0.79, 1.31)	0.775
	M	0.95 (0.64, 1.40)		0.94 (0.65, 1.38)		0.95 (0.66, 1.37)		1.13 (0.85, 1.51)	
≥21 to <25 °C	F	2.44 (2.01, 2.94)		2.44 (2.01, 2.94)		2.44 (2.01, 2.94)		1.36 (1.15, 1.60)	
	M	2.29 (1.82, 2.92)		2.27 (1.82, 2.83)		2.27 (1.82, 2.83)		1.25 (1.00, 1.54)	
≥25 °C	F	0.52 (0.44, 0.63)		0.52 (0.44, 0.63)		0.52 (0.44, 0.63)		0.84 (0.75, 0.95)	
	M	0.53 (0.42, 0.66)		0.53 (0.44, 0.66)		0.54 (0.44, 0.66)		0.87 (0.71, 1.09)	
Urine chloride concentration (mmol/l)									
<24 °C	F	0.99 (0.95, 1.04)	0.809	0.99 (0.94, 1.04)	0.778	0.99 (0.94, 1.04)	0.768	0.90 (0.84, 0.95)	0.729
	M	1.01 (0.95, 1.07)		1.01 (0.95, 1.07)		1.01 (0.95, 1.06)		0.92 (0.86, 0.98)	
≥24 °C	F	1.19 (1.08, 1.30)		1.20 (1.09, 1.31)		1.20 (1.11, 1.31)		1.15 (1.03, 1.28)	
	M	1.19 (1.09, 1.27)		1.19 (1.09, 1.27)		1.19 (1.11, 1.27)		1.13 (1.04, 1.23)	

F, female; M, male.

Regression co-efficient and 95% confidence intervals represent geomatric mean ratio in relation to 5 ℃ increase in average ambient temperature.

Level of significance is 0.004 (α = 0.05/12 or 0.004).

*P*-value indicates the overall significance of average temperature and sex interaction for all segments of ambient temperature using the Wald test.

**Table 3:** Piecewise linear regression model showing associations between daily average temperature and 24-hour excretion of urine biomarkers for different average temperature segments using individual, household, and community level random intercepts

Temperature segments	Sex	Model 1	*P*-value	Model 2	*P*-value	Model 3	*P*-value	Model 4	*P*-value
Urine total protein excretion (mg/24h)^a^									
<23 °C	F	1.19 (1.00, 1.45)	0.939	1.19 (0.98, 1.45)	0.444	1.19 (0.98, 1.43)	0.434	1.16 (0.90, 1.49)	0.274
	M	1.23 (1.00, 1.51)		1.20 (0.96, 1.48)		1.20 (0.96, 1.48)		1.15 (0.88, 1.51)	
≥23 °C	F	0.57 (0.52, 0.63)		0.57 (0.52, 0.63)		0.57 (0.52, 0.63)		0.64 (0.54, 0.77)	
	M	0.57 (0.52, 0.63)		0.59 (0.54, 0.64)		0.59 (0.54, 0.64)		0.68 (0.58, 0.79)	
Urine magnesium excretion (mmol/24h)^a^									
<21 °C	F	1.31 (1.03, 1.67)	0.235	1.28 (1.02, 1.62)	0.015	1.27 (1.01, 1.62)	0.023	1.27 (0.90, 1.79)	0.483
	M	1.13 (0.89, 1.42)		1.09 (0.85, 1.42)		1.09 (0.85, 1.42)		1.12 (0.77, 1.62)	
≥21 to <26 °C	F	1.15 (1.01, 1.32)		1.16 (1.01, 1.34)		1.16 (1.02, 1.34)		0.83 (0.70, 0.98)	
	M	1.21 (1.01, 1.45)		1.19 (1.00, 1.43)		1.20 (1.00, 1.43)		0.85 (0.66, 1.09)	
≥26 °C	F	1.11 (0.96, 1.27)		1.11 (0.96, 1.27)		1.09 (0.96, 1.27)		0.77 (0.59, 1.01)	
	M	1.13 (0.92, 1.39)		1.18 (0.96, 1.46)		1.20 (0.97, 1.46)		0.81 (0.58, 1.15)	
Urine calcium excretion (mmol/24h)^a^									
<21 °C	F	1.00 (0.70, 1.45)	0.725	0.98 (0.70, 1.42)	0.679	0.97 (0.67, 1.42)	0.658	1.15 (0.87, 1.51)	0.626
	M	1.07 (0.70, 1.63)		1.02 (0.70, 1.52)		1.02 (0.68, 1.52)		1.25 (0.90, 1.73)	
≥21 to <25 °C	F	2.20 (1.82, 2.66)		2.23 (1.86, 2.69)		2.23 (1.86, 2.69)		1.32 (1.14, 1.55)	
	M	2.08 (1.63, 2.61)		2.05 (1.65, 2.53)		2.05 (1.65, 2.53)		1.20 (1.01, 1.42)	
≥25 °C	F	0.41 (0.33, 0.51)		0.41 (0.33, 0.50)		0.41 (0.33, 0.50)		0.72 (0.61, 0.85)	
	M	0.41 (0.32, 0.53)		0.43 (0.35, 0.54)		0.44 (0.35, 0.54)		0.78 (0.61, 0.99)	
Following variables were not log-transformed									
Urine Sodium excretion (mmol/24h)^b^									
<21 °C	F	5.09 (−18.23, 28.41)	0.849	1.67 (−22.60, 25.95)	0.979	1.11 (−23.10, 25.33)	0.976	4.98 (−18.54, 28.51)	0.959
	M	9.35 (−13.43, 32.13)		1.45 (−20.31, 23.22)		1.28 (−20.56, 23.11)		6.16 (−15.03, 27.35)	
≥21 °C	F	−5.02 (−12.17, 2.14)		−4.09 (−11.12, 2.95)		−4.07 (−11.05, 2.91)		−7.69 (−17.33, 1.95)	
	M	−5.96 (−13.05, 1.13)		−3.38 (−10.67, 3.90)		−3.34 (−10.67, 3.99)		−6.98 (−15.43, 1.48)	
Urine Potassium excretion (mmol/24h)^b^									
<25 °C	F	1.33 (−0.88, 3.54)	0.078	1.15 (−1.17, 3.48)	0.056	1.11 (−1.20, 3.42)	0.053	0.33 (−2.12, 2.79)	0.032
	M	3.30 (2.32, 4.27)		2.71 (1.33, 4.08)		2.69 (1.33, 4.04)		2.02 (0.35, 3.69)	
≥25 °C	F	−6.86 (−9.38, −4.34)		−6.44 (−9.36, −3.52)		−6.40 (−9.32, −3.47)		−2.61 (−6.23, 1.01)	
	M	−7.11 (−9.19, −4.23)		−5.55 (−8.95, −2.16)		−5.51 (−8.92, −2.11)		−1.63 (−4.91, 1.64)	
Urine Chloride excretion (mmol/24h)^b^									
<21 °C	F	−7.59 (−31.63, 16.46)	0.768	−11.20 (−35.90, 13.49)	0.966	−11.79 (−36.46, 12.88)	0.963	−5.35 (−26.54, 15.83)	0.889
	M	−4.52 (−24.72, 15.67)		−10.30 (−29.40, 8.81)		−10.29 (−29.67, 9.09)		−0.58 (−17.98, 16.81)	
≥21 to <25 °C	F	2.45 (−7.59, 12.48)		3.73 (−7.09, 14.56)		3.73 (−7.22, 14.68)		−14.71 (−30.41, 0.99)	
	M	7.36 (−2.39, 17.11)		6.44 (−2.83, 15.71)		6.28 (−3.03, 15.58)		−13.81 (−27.12, −0.51)	
≥25 °C	F	−11.49 (−25.63, 2.64)		−10.45 (−25.04, 4.14)		−10.41 (−24.99, 4.16)		1.61 (−15.68, 18.89)	
	M	−15.68 (−29.29, −2.08)		−10.09 (−24.61, 4.42)		−9.87 (−24.42, 4.67)		3.38 (−13.25, 20.01)	
Urine volume (liter/24h)^b^									
<22 °C	F	0.19 (0.07, 0.32)	0.921	0.19 (0.07, 0.32)	0.906	0.19 (0.07, 0.32)	0.911	0.29 (0.08, 0.49)	0.992
	M	0.20 (0.05, 0.34)		0.19 (0.05, 0.33)		0.19 (0.06, 0.33)		0.30 (0.11, 0.48)	
≥22 °C	F	−0.35 (−0.40, −0.30)		−0.35 (−0.40, −0.31)		−0.35 (−0.40, −0.31)		−0.21 (−0.30, −0.11)	
	M	−0.36 (−0.42, −0.31)		−0.37 (−0.42, −0.31)		−0.37 (−0.42, −0.31)		−0.21 (−0.32, −0.10)	

F, female; M, male.

Level of significance is 0.004 (α = 0.05/12 or 0.004).

*P*-value indicates the overall significance of average temperature and sex interaction for all segments of ambient temperature using the Wald test.

a Regression eo-efficient and 95% confidence intervals represent geometric mean ratio in relation to 5 ℃ increase in average ambient temperature.

b Indicates change in mean biomarkers due to 5 ℃ increases in average ambient tempearture.

Moreover, the RCS plots generated from the analyses involving participants without comorbidities and with complete 24- hour urine collection demonstrated similar relationships between ambient temperature and urinary electrolytes, 24-hour urine total protein, and volume as illustrated in the Supplementary Figures S13 and S14. Stratified analyses among women aged ≤49 and >49 years exhibited similar associations between average ambient temperatures and urine outcomes (Supplementary Figure S15).

## Discussion

Our findings revealed no discernible statistical difference between men and women in the relationships observed between ambient temperature and various urine biomarkers. In contrast to many epidemiological studies highlighting men as particularly vulnerable to high ambient heat, our results suggest that women are equally susceptible to ambient temperature-related urinary electrolytes and other biomarkers changes. We found higher urine sodium and chloride concentrations but their lower 24-hour excretions for both sexes when ambient temperature increased, which is consistent with previous evidence and suggest substantial sweating by both men and women.^19^

High ambient temperature was associated with higher urinary concentrations and 24-hour excretions of calcium and magnesium for both men and women. Sunlight exposure increases vitamin D production, triggering calcium excretion via urine.^55^^,^^56^ High urine calcium excretion during a hot ambient environment coupled with low urine volume may cause supersaturation of calcium salts, increasing the risk of calcium stones formation. The prevalence of calcium kidney stones was reportedly higher in tropical and subtropical regions,^9^^,^^10^^,^^57^, ^58^, ^59^ and this aligned with our findings. Several studies in the United States have reported that men had a higher prevalence of kidney stones;^17^^,^^50^ however, other evidence supports no sex-specific difference in kidney stone prevalence.^57^ Moreover, recent findings on growing kidney stone incidence in women and adolescent girls,^60^ suggest a heightened susceptibility to ambient heat exposure among women.

Our study aimed to collect 24-hour urine volume as an objective measurement of daily water intake from all sources. Because daily water intake not only depends on fluid or beverage consumption but also comes from food, relying on self-reported daily water intake may lead to inaccurate estimation and misclassification due to recall bias.^61^ We found high ambient tempeartures associated with lower urine volume for both men and women.

In contrast to our findings, a laboratory-based study in the United States examining 28,498 urine samples highlighted that men had remarkably lower 24-hour urine volume than women during summer.^19^ Nevertheless, a hospital-based study in the United States with 136 patients with a history of nephrolithiasis failed to demonstrate significant seasonal variation in 24-hour urine volume, where sex appeared to have no impact.^62^ In both studies, participants socioeconomic, cultural, and behavioral factors were not considered in the analyses. The possible explanation of the association between ambient temperature and lower 24-hour excretion of urine total protein is temperature-induced lower blood pressure.^63^ Proteinuria is sensitive to glomerular hypertension and is a marker of renal function; thus, reduced glomerular pressure during nonextreme high ambient temperature may alleviate proteinuria by blocking the renin-angiotensin-aldosterone system.^64^, ^65^, ^66^

Several studies have highlighted the presence of sex-specific differences in physiologic responses or thermoregulation and temperature-dependent kidney function.^17^^,^^18^ Thermoregulation is a complex process of physical, chemical, and behavioral responses essential in regulating body temperature within a restricted range.^67^ Men mostly depend on the evaporative process, whereas women rely on convection for body temperature loss.^68^ Evaporation is a process of heat loss to the environment as atmospheric water vapor is released from the skin and respiratory tract.^69^ Convection is the transfer of heat caused by increased body movement in air currents or water.^17^^,^^69^ Higher metabolic heat production in response to atmospheric heat exposure among men causes high evaporative heat loss through sweating.^17^^,^^70^

Therefore, men can lose more water and electrolytes, such as sodium and chloride, through sweating. Moreover, dimorphic renal physiology results in more concentrated urine in men because of greater renal tubular body water reabsorption.^71^^,^^72^ Conversely, women have lower sweat production per gland than men and generally transfer body heat from the skin surface to its surrounding air currents by convective heat exchange.^17^^,^^67^^,^^73^ However, when core body tempearture increases due to higher ambient tempearture, convention alone cannot reduce body temperature.^74^ Higher body mass and subcutaneous fat in women additionally interrupt heat exchange from the female body.^17^^,^^75^ In such a situation, evaporation appears to be the only key mechanism for heat dissipation and body temperature regulation in women.^74^

The lack of sex-differentiated physiologic responses to ambient temperature in our study may be due to environmental, socioeconomic, cultural, and behavioral factors that may expose both men and women equally to high ambient temperature.^17^^,^^67^^,^^69^ Typically, in rural coastal Bangladesh, men work outside in agricultural fields and fishing-related occupations under direct exposure of atmospheric heat and perform extended hours of work with inadequate hydration. Women also participate in agricultural and farming work in the backyard gardens or lands surrounding their homesteads,^20^ in addition to their household chores. Both these occupational exposures require substantial physical activity, causing sweating, including water and electrolyte loss.

Furthermore, housing structures in rural Bangladesh, including the roofs, are mostly made of corrugated tin sheets, a heat-trapping material that keeps elevating indoor temperature during daytime,^76^ exposing women to severe atmospheric heat. In addition, women in this cultural setting wear long clothes covering most of the body surfaces that can restrict heat dissipation through skin.^17^^,^^68^ Therefore, the heat exposure of women in this context, both indoors and outdoors, considering environmental, sociobehavioral and cultural factors, may trigger thermoregulatory responses making them equally vulnerable to ambient temperature as men.

We acknowledge several limitations in this study. First, we used weather-station-based temperature data, often regarded as a gold standard.^77^ The temperature data in this study were collected from the Bangladesh Meteorological Department, the official source of meteorological and climate data in Bangladesh. There are 34 meteorological stations monitored across the country.^78^ Although station-based data are considered representative of actual ambient conditions, lack of precision in spatial exposure variability may result from the sparse density of meteorological stations,^79^ particularly for households far from weather stations. Such inaccuracy in exposure measurement may cause measurement error, thereby can introduce exposure misclassification. Collecting meteorological data at residential locations is often very expensive. Therefore, ambient temperature data from the weather station is a routine measure in environmental and occupational epidemiology research in Bangladesh and elsewhere.^80^ Daily meteorological data can also be obtained from spatially gridded dataset; however, such data were not available for the study region during the time of study.

Second, we lacked data on biomarkers that include sweat (electrolytes), blood (electrolytes), hormonal (aldosterone, arginine, vasopressin), and functional (estimated glomerular filtration rate) parameters of kidney damage in response to ambient heat. These biomarkers can help to understand whether sex has an effect-modifying role on ambient heat-related nephropathies. We used objective markers of hypohydration such as 24-hour urine volume and urine sodium and chloride concentrations.^81^ However, we did not collect biomarkers of hydration status such as serum or urine osmolality, which are good indicators of acute dehydration,^82^^,^^83^ but may lack sensitivity in detecting mild hypohydration.^84^

Third, 24-hour urine biomarker excretions can be affected by overcollection or undercollection in nonclinical settings.^49^ It is recommended to use para-aminobenzoic acid in epidemiological studies to measure the completeness of 24-hour urine collection;^85^ however, our study did not have this component. Nevertheless, creatinine is frequently used as a marker for complete 24-hour urine collection through indirect approaches^49^; and we used such approach to evaluate completeness of 24-hour urine collection. We performed supplemental analyses among participants with complete 24-hour urine collection.

Fourth, these results may not be generalizable to other noncoastal geographic areas in Bangladesh due to the different geographic and climactic diversity which can hugely impact community livelihood, food security, and water resources protection.^86^^,^^87^ Because our investigation was limited to 5 months only prior to the monsoon season in Bangladesh, we were unable to investigate the effect of extreme temperature variations along with joint effect of monsoon season on sex-specific associations between ambient temperature and outcome variables.

There are several strengths of this study. The large sample size and the stepped-wedged cluster experimental design are the strengths of this study. Repeated measures of 24-hour urine electrolytes, total protein, and volume for 5 consecutive visits, spanning both hot and cold seasons is also a strength, especially, given the observed intraindividual variations of the 24-hour urinary biomarkers' excretion. Therefore, repeated measurements provide more accurate estimation than a single 24-hour measurement or spot urine sample.^88^^,^^89^ Using statistical methods such as random intercepts for the individual, household, and community levels in statistical analyses helps in controlling unmeasured nontime-varying confounders, minimizing bias in our estimates. We believe that this study’s findings are relevant to other coastal populations with similar coastal and climatic contexts, including saltwater intrusion affected areas where communities experience drinking water salinity (e.g., Ganges River delta, Mekong, and Red River delta).

## Conclusion

Our analyses provide fundamental pathophysiological insights into temperature-induced kidney disease burden by looking at routine urine biomarkers. The study findings suggest that there are no sex differences in ambient temperature-dependent hypohydration, urine electrolyte, and 24-hour urine protein excretions. Therefore, women are equally susceptible to ambient heat-induced changes in urinary markers, as men. Further research is warranted by measuring individual-level temperature exposure at participants' residences and collecting hypohydration and electrolyte markers in blood and sweat to better understand sex-specific vulnerabilities due to ambient temperature.

## Disclosure

All the authors declared no conflicting interests.

## Supplementary Materials

Supplementary File (PDF)

**Figure S1.** Map of the study area (Khulna, Satkhira, and Bagerhat) in southwest coastal Bangladesh.

**Figure S2.** Histogram of the concentration and 24-hour excretion of urinary electrolytes (sodium, potassium, chloride, calcium, and magnesium), including 24-hour excretion of urine total protein and volume.

**Figure S3.** Relationship between ambient temperature (minimum, average, and maximum) and 24-hour urine volume for males and females.

**Figure S4.** Relationship between ambient temperature (minimum, average, and maximum) and 24-hour urine volume for different age groups.

**Figure S5.** Violin plots illustrating the concentration of urine electrolytes (sodium, potassium, and chloride) for males and females across tertiles of ambient temperature (minimum ambient temperature: tertile 1: ≤15.6 °C , tertile 2: 15.7–19.5 °C, tertile 3: ≥19.6 °C; average ambient temperature: tertile 1: ≤21.2 °C, tertile 2: 21.3–24.9 °C, tertile 3: ≥25 °C; maximum ambient temperature: tertile 1: ≤28.4 °C, tertile 2: 28.5–31 °C, tertile 3: ≥31.1 °C).

**Figure S6.** Violin plots illustrating the concentration of urine electrolytes (calcium and magnesium) for males and females across tertiles of ambient temperature (minimum ambient temperature: tertile 1 : ≤15.6 °C, tertile 2: 15.7–19.5 °C, tertile 3: ≥19.6 °C; average ambient temperature: tertile 1: ≤21.2 °C, tertile 2: 21.3–24.9 °C, tertile 3: ≥25 °C; maximum ambient temperature: tertile 1: ≤28.4 °C, tertile 2: 28.5–31 °C, tertile 3: ≥31.1 °C).

**Figure S7.** Violin plots illustrating 24-hour excretion of urine electrolytes (sodium, potassium, and chloride) for males and females across tertiles of ambient temperature (minimum ambient temperature: tertile 1: ≤15.6 °C, tertile 2: 15.7–19.5 °C, tertile 3: ≥19.6 °C ; average ambient temperature: tertile 1: ≤21.2 °C, tertile 2: 21.3–24.9 °C, tertile 3: ≥25 °C ; maximum ambient temperature: tertile 1: ≤28.4 °C, tertile 2: 28.5–31 °C, tertile 3: ≥31.1 °C).

**Figure S8.** Violin plots illustrating 24-hour excretion of urine electrolytes (calcium and magnesium) for males and females across tertiles of ambient temperature (minimum ambient temperature: tertile 1: ≤15.6 °C, tertile 2: 15.7–19.5 °C, tertile 3: ≥19.6 °C; average ambient temperature: tertile 1: ≤21.2 °C, tertile 2: 21.3–24.9 °C, tertile 3: ≥25 °C; maximum ambient temperature: tertile 1 : ≤28.4 °C, tertile 2: 28.5–31 °C, tertile 3: ≥31.1 °C).

**Figure S9.** Violin plots illustrating 24-hour excretion of urine total protein for males and females across tertiles of ambient temperature (minimum ambient temperature: tertile 1 : ≤15.6 °C, tertile 2: 15.7–19.5 °C, tertile 3: ≥19.6 °C; average ambient temperature: tertile 1 : ≤21.2 °C, tertile 2: 21.3–24.9 °C, tertile 3: ≥25 °C; maximum ambient temperature: tertile 1 : ≤28.4 °C, tertile 2: 28.5–31 °C, tertile 3: ≥31.1 °C).

**Figure S10.** Restricted cubic spline plots with their 95% confidence bands using linear mixed-effect model demonstrating sex-stratified association between the concentration and 24-hour excretion of urinary electrolytes (sodium, potassium, chloride, calcium, and magnesium) and 24-hour excretion of urine total protein and volume with minimum ambient temperature, adjusted for age, body mass index, physical exercise, smoking, alcohol consumption, sleep duration, religion, household wealth, time of visit, drinking water salinity, and humidity.

**Figure S11.** Restricted cubic spline plots with their 95% confidence bands using linear mixed-effect model demonstrating sex-stratified association between the concentration and 24-hour excretion of urinary electrolytes (sodium, potassium, chloride, calcium, and magnesium) and 24-hour excretion of urine total protein and volume with maximum ambient temperature, adjusted for age, body mass index, physical exercise, smoking, alcohol consumption, sleep duration, religion, household wealth, time of visit, drinking water salinity, and humidity.

**Figure S12.** Restricted cubic spline plots with their 95% confidence bands using linear mixed-effect model demonstrating sex-stratified association of average ambient temperature with urine electrolytes, 24-hour urine total protein, and volume among participants who did not report any comorbidities after adjusting for age, body mass index, physical exercise, smoking, alcohol consumption, sleep duration, religion, household wealth, time of visit, drinking water salinity, and humidity.

**Figure S13.** Restricted cubic spline plots with their 95% confidence bands using a linear mixed-effect model demonstrating sex-stratified associations between the concentration and 24-hour excretion of urinary electrolytes (sodium, potassium, chloride, calcium, and magnesium) and 24-hour urine total protein and volume with average ambient temperature among participants with complete 24-hour urine collection based on creatinine index >0.7. The restricted cubic spline plots were adjusted for age, body mass index, physical exercise, smoking, alcohol consumption, sleep duration, religion, household wealth, time of visit, drinking water salinity, and humidity.

**Figure S14.** Restricted cubic spline plots with their 95% confidence bands using linear mixed-effect model demonstrating sex-stratified association between average temperature and urine electrolytes and total protein after excluding outliers with adjusting for age, body mass index, physical exercise, smoking, alcohol consumption, sleep duration, religion, household wealth, time of visit, drinking water salinity, and humidity.

**Figure S15.** Restricted cubic spline plots with their 95% confidence bands using linear mixed-effect model demonstrating age-stratified association among women, between the concentration and 24-hour excretion of urinary electrolytes (sodium, potassium, chloride, calcium, and magnesium) and 24-hour excretion of urine total protein and urine volume with average ambient temperature, adjusted for other covariates.

**Table S1.** Piecewise linear regression model showing associations between maximum temperature and concentrations of urine biomarkers for different maximum temperature segments using individual, household, and community level random intercepts. *P*-value indicates the overall significance of maximum temperature and sex interaction for all segments of ambient temperature using the Wald test.

**Table S2.** Piecewise linear regression model showing associations between maximum temperature and 24-hour excretion of urine biomarkers for different maximum temperature segments using individual, household, and community level random intercepts. *P*-value indicates the overall significance of maximum temperature and sex interaction for all segments of ambient temperature using the Wald test.

**Table S3.** Piecewise linear regression model showing associations between minimum temperature and concentrations of urine biomarkers for different minimum temperature segments using individual, household, and community level random intercepts. *P*-value indicates the overall significance of minimum temperature and sex interaction for all segments of ambient temperature using the Wald test.

**Table S4.** Piecewise linear regression model showing associations between minimum temperature and 24-hour excretion of urine biomarkers for different minimum temperature segments using individual, household, and community level random intercepts. *P*-value indicates the overall significance of minimum temperature and sex interaction for all segments of ambient temperature using the Wald test.

**Table S5.** Piecewise linear quantile model showing association between daily average temperature and the concentration of urine biomarkers for different average temperature segments using individual level random intercepts. *P*-value indicates the overall significance of average temperature and sex interaction for all segments of ambient temperature using the Wald test.

**Table S6.** Piecewise linear quantile model showing association between daily average temperature and the 24-hour urine biomarkers for different average temperature segments using individual level random intercepts. *P*-value indicates the overall significance of average temperature and sex interaction for all segments of ambient temperature using the Wald test.

**STROBE Statement.**
