# Supplementary material for: Sex-Specific Association of Ambient Temperature With Urine Biomarkers in Southwest Coastal Bangladesh
Source: Kidney Int Rep. 2024 Mar 11;9(6):1860–75. doi: 10.1016/j.ekir.2024.03.002 (PMC11184407; doi:10.1016/j.ekir.2024.03.002)
Supplement: Supplementary File (PDF) [file mmc1.pdf]

**Supplementary materials**

# Supplementary tables

**Table S1:** Piecewise linear regression model showing associations between maximum temperature and concentrations of urine biomarkers for different maximum temperature segments using individual, household, and community level random intercepts. P-value indicates the overall significance of maximum temperature and sex interaction for all segments of ambient temperature using the Wald test. Regression co-efficient and 95% confidence intervals represent geometric mean ratio in relation to 5℃ increases in maximum ambient temperature.

| Temperature segments | Sex | Model 1 | p-value | Model 2 | p-value | Model 3 | p-value | Model 4 | p-value |
|---|---|---|---|---|---|---|---|---|---|
| **Urine Sodium concentration (mmol/L)** | | | | | | | | | |
| <32 °C | F | 1.05 (1.01, 1.09) | 0.819 | 1.05 (1.01, 1.09) | 0.791 | 1.05 (1.01, 1.09) | 0.811 | 0.97 (0.93, 1.02) | 0.730 |
| | M | 1.06 (1.02, 1.09) | | 1.05 (1.02, 1.08) | | 1.05 (1.02, 1.09) | | 0.97 (0.93, 1.01) | |
| ≥32 °C | F | 1.31 (1.09, 1.54) | | 1.31 (1.09, 1.55) | | 1.31 (1.09, 1.55) | | 1.16 (0.96, 1.39) | |
| | M | 1.25 (1.09, 1.39) | | 1.25 (1.11, 1.39) | | 1.25 (1.11, 1.40) | | 1.09 (0.97, 1.26) | |
| **Urine Potassium concentration (mmol/L)** | | | | | | | | | |
| <27 °C | F | 0.78 (0.71, 0.86) | 0.293 | 0.79 (0.71, 0.87) | 0.334 | 0.79 (0.70, 0.87) | 0.314 | 0.90 (0.79, 1.02) | 0.278 |
| | M | 0.84 (0.74, 0.98) | | 0.84 (0.74, 0.97) | | 0.85 (0.74, 0.97) | | 0.99 (0.84, 1.16) | |
| ≥27-<32 °C | F | 1.17 (1.08, 1.27) | | 1.17 (1.08, 1.27) | | 1.17 (1.08, 1.27) | | 1.03 (0.93, 1.14) | |
| | M | 1.22 (1.13, 1.32) | | 1.21 (1.13, 1.31) | | 1.21 (1.12, 1.31) | | 1.06 (0.96, 1.16) | |
| ≥32 °C | F | 2.56 (2.20, 3.10) | | 0.95 (0.79, 1.13) | | 0.95 (0.79, 1.13) | | 1.01 (0.84, 1.22) | |
| | M | 0.98 (0.81, 1.17) | | 0.99 (0.82, 1.19) | | 0.99 (0.82, 1.19) | | 1.05 (0.90, 1.22) | |
| **Urine Magnesium concentration (mmol/L)** | | | | | | | | | |
| <28 °C | F | 1.03 (0.83, 1.30) | 0.049 | 1.03 (0.83, 1.30) | 0.046 | 1.03 (0.83, 1.30) | 0.042 | 1.22 (0.88, 1.72) | 0.131 |
| | M | 0.84 (0.67, 1.05) | | 0.84 (0.67, 1.06) | | 0.84 (0.67, 1.06) | | 0.99 (0.71, 1.38) | |
| ≥28-<32 °C | F | 1.46 (1.85, 1.80) | | 1.46 (1.17, 1.80) | | 1.46 (1.17, 1.80) | | 0.77 (0.61, 0.97) | |
| | M | 1.57 (1.30, 1.90) | | 1.57 (1.28, 1.90) | | 1.55 (1.27, 1.90) | | 0.83 (0.66, 1.04) | |
| ≥32 °C | F | 1.42 (1.14, 1.79) | | 1.43 (1.15, 1.79) | | 1.43 (1.15, 1.79) | | 0.98 (0.76, 1.26) | |
| | M | 1.55 (1.27, 1.90) | | 1.57 (1.27, 1.93) | | 1.58 (1.28, 1.97) | | 1.04 (0.79, 1.34) | |
| **Urine Calcium Concentration (mmol/L)** | | | | | | | | | |
| <31 °C | F | 1.68 (1.49, 1.90) | 0.914 | 1.68 (1.49, 1.90) | 0.979 | 1.68 (1.49, 1.90) | 0.983 | 1.26 (1.14, 1.40) | 0.929 |
| | M | 1.70 (1.46, 1.97) | | 1.70 (1.46, 1.95) | | 1.68 (1.46, 1.95) | | 1.23 (1.12, 1.38) | |
| ≥31 °C | F | 0.63 (0.51, 0.79) | | 0.63 (0.51, 0.79) | | 0.63 (0.50, 0.79) | | 0.81 (0.65, 1.00) | |
| | M | 0.61 (0.50, 0.74) | | 0.62 (0.52, 0.74) | | 0.62 (0.52, 0.74) | | 0.83 (0.70, 0.97) | |
| **Urine Chloride concentration (mmol/L)** | | | | | | | | | |
| <30 °C | F | 0.99 (1.06, 1.04) | 0.861 | 0.99 (0.94, 1.04) | 0.870 | 0.99 (0.89, 0.98) | 0.870 | 0.93 (0.89, 0.98) | 0.817 |
| | M | 1.01 (0.95, 1.06) | | 1.00 (0.95, 1.06) | | 0.94 (0.90, 0.99) | | 0.94 (0.90, 0.99) | |
| ≥30 °C | F | 1.23 (1.12, 1.38) | | 1.25 (1.12, 1.38) | | 1.09 (0.98, 1.23) | | 1.09 (0.98, 1.23) | |
| | M | 1.22 (1.12, 1.32) | | 1.21 (1.13, 1.31) | | 1.07 (0.99, 1.16) | | 1.07 (0.99, 1.16) | |

**Footnote:** Level of significance is 0.004 ($\alpha = 0.05/12$ or 0.004)

**Table S2**: Piecewise linear regression model showing associations between maximum temperature and 24-hour excretion of urine biomarkers for different maximum temperature segments using individual, household, and community level random intercepts. P-value indicates the overall significance of maximum temperature and sex interaction for all segments of ambient temperature using the Wald test.

| Temperature segments | Sex category | Model 1 | p-value | Model 2 | p-value | Model 3 | p-value | Model 4 | p-value |
|---|---|---|---|---|---|---|---|---|---|
| **Urine total protein excretion (mg/24 hour) $** | | | | | | | | | |
| <32 °C | F | 0.88 (0.83, 0.93) | 0.412 | 0.88 (0.83, 0.93) | 0.359 | 0.88 (0.83, 0.93) | 0.355 | 0.91 (0.84, 0.99) | 0.286 |
| | M | 0.84 (0.78, 0.91) | | 0.84 (0.78, 0.91) | | 0.84 (0.78, 0.91) | | 0.87 (0.79, 0.95) | |
| ≥32°C | F | 0.29 (0.25, 0.34) | | 0.29 (0.24, 0.34) | | 0.29 (0.24, 0.34) | | 0.28 (0.23, 0.33) | |
| | M | 0.34 (0.29, 0.41) | | 0.34 (0.29, 0.41) | | 0.34 (0.28, 0.41) | | 0.33 (0.27, 0.41) | |
| **Urine Magnesium excretion (mmol/24 hour) $** | | | | | | | | | |
| <28 °C | F | 1.19 (1.03, 1.38) | 0.664 | 1.19 (1.02, 1.36) | 0.729 | 1.17 (1.02, 1.36) | 0.736 | 1.28 (1.09, 1.51) | 0.721 |
| | M | 1.07 (0.90, 1.30) | | 1.08 (0.90, 1.30) | | 1.08 (0.90, 1.30) | | 1.17 (0.97, 1.42) | |
| ≥28°C | F | 1.19 (1.12, 1.26) | | 1.19 (1.12, 1.27) | | 1.19 (1.12, 1.27) | | 0.81 (0.74, 0.90) | |
| | M | 1.23 (1.14, 1.32) | | 1.22 (1.14, 1.34) | | 1.22 (1.14, 1.32) | | 0.84 (0.75, 0.92) | |
| **Urine Calcium excretion (mmol/24 hour) $** | | | | | | | | | |
| <31 °C | F | 1.63 (1.52, 1.75) | 0.875 | 1.63 (1.52, 1.75) | 0.879 | 1.63 (1.52, 1.75) | 0.886 | 1.32 (1.22, 1.43) | 0.960 |
| | M | 1.65 (1.51, 1.79) | | 1.63 (1.49, 1.79) | | 1.63 (1.49, 1.79) | | 1.30 (1.19, 1.43) | |
| ≥31 °C | F | 0.46 (0.41, 0.52) | | 0.47 (0.42, 0.52) | | 0.47 (0.42, 0.52) | | 0.75 (0.65, 0.85) | |
| | M | 0.44 (0.39, 0.51) | | 0.45 (0.39, 0.51) | | 0.45 (0.39, 0.51) | | 0.75 (0.64, 0.87) | |
| **Following variables were not log- transformed** | | | | | | | | | |
| **Urine Sodium excretion (mmol/24 hour) ¥** | | | | | | | | | |
| <28 °C | F | 1.32 (-11.02, 13.67) | 0.267 | 0.55 (-11.80, 12.90) | 0.191 | 0.08 (-12.26, 12.43) | 0.189 | 4.91 (-8.33, 18.16) | 0.189 |
| | M | 15.51 (0.12, 30.90) | | 14.99 (-0.37, 30.36) | | 14.72 (-0.64, 30.08) | | 20.80 (4.59, 37.00) | |
| ≥28 °C | F | -2.98 (-8.24, 2.27) | | -2.52 (-7.80, 2.76) | | -2.47 (-7.74, 2.81) | | -2.26 (-10.34, 5.83) | |
| | M | -8.80 (-15.08, -2.52) | | -9.52 (-15.81, -3.24) | | -9.43 (-15.71, -3.15) | | -8.78 (-17.59, 0.02) | |
| **Urine Chloride excretion (mmol/24 hour) ¥** | | | | | | | | | |
| <30 °C | F | -1.02 (-8.72, 6.68) | 0.483 | -1.39 (-9.11, 6.33) | 0.374 | -1.58 (-9.30, 6.14) | 0.380 | -5.28 (-14.14, 3.59) | 0.386 |
| | M | 5.50 (-4.05, 15.05) | | 4.80 (-4.76, 14.37) | | 4.69 (-4.87, 14.25) | | 1.82 (-8.83, 12.47) | |
| ≥30 °C | F | -6.08 (-14.12, 1.95) | | -4.85 (-12.95, 3.24) | | -4.82 (-12.91, 3.27) | | 2.22 (-8.65, 13.08) | |
| | M | -12.76 (-22.34, -3.19) | | -13.47 (-23.06, -3.87) | | -13.32 (-22.91, -3.72) | | -5.75 (-17.90, 6.40) | |
| **Urine Potassium excretion (mmol/24 hour) ¥** | | | | | | | | | |
| <27 °C | F | -5.12 (-9.79, -0.45) | 0.028 | -5.18 (-9.87, -0.49) | 0.036 | -5.42 (-10.11, -0.73) | 0.032 | -0.82 (-5.81, 4.18) | 0.019 |
| | M | 3.99 (-1.79, 9.78) | | 4.21 ( -1.58, 9.99) | | 4.12 (-1.67, 9.90) | | 9.03 (2.99, 15.06) | |
| ≥27-<31 °C | F | 4.06 (1.95, 6.17) | | 3.99 (1.86, 6.12) | | 4.07 (1.94, 6.19) | | 2.03 (-0.72, 4.79) | |
| | M | 3.56 (0.99, 6.12) | | 3.19 (0.62, 5.77) | | 3.19 (0.61, 5.76) | | 1.21 (-1.90, 4.32) | |
| ≥31 °C | F | -8.65 (-10.83, -6.48) | | -8.36 (-10.56, -6.17) | | -8.42 (-10.61, -6.22) | | -2.35 (-5.04, 0.33) | |
| | M | -7.70 (-10.26, -5.13) | | -7.53 (-10.10, -4.95) | | -7.48 (-10.06, -4.91) | | -1.21 (-4.26, 1.84) | |
| **Urine volume (liter/24 hour) ¥** | | | | | | | | | |
| <30 °C | F | 0.06 (-0.02, 0.13) | 0.684 | 0.05 (-0.02, 0.12) | 0.641 | 0.05 (-0.02, 0.12) | 0.645 | 0.13 (0.05, 0.22) | 0.763 |
| | M | 0.07 (-0.02, 0.16) | | 0.07 (-0.02, 0.16) | | 0.07 (-0.02, 0.16) | | 0.17 (0.07, 0.27) | |
| ≥30 °C | F | -0.48 (-0.55, -0.40) | | -0.48 (-0.55, -0.40) | | -0.48 (-0.55, -0.40) | | -0.18 (-0.29, -0.08) | |
| | M | -0.53 (-0.62, -0.44) | | -0.53 (-0.62, -0.44) | | -0.53 (-0.62, -0.44) | | -0.23 (-0.34, -0.11) | |

**Footnote:** Level of significance is 0.004 ($\alpha = 0.05/12$ or 0.004)

$ Regression co-efficient and 95% confidence intervals represent geometric mean ratio in relation to 5℃ increases in maximum ambient temperature.

¥ Indicate change in mean biomarkers due to 5℃ increases in maximum ambient temperature.

**Table S3:** Piecewise linear regression model showing associations between minimum temperature and concentrations of urine biomarkers for different minimum temperature segments using individual, household, and community level random intercepts. P-value indicates the overall significance of minimum temperature and sex interaction for all segments of ambient temperature using the Wald test. Regression co-efficient and 95% confidence intervals represent geometric mean ratio in relation to 5℃ increases in minimum ambient temperature.

| Temperature segments | Sex | Model 1 | p-value | Model 2 | p-value | Model 3 | p-value | Model 4 | p-value |
|---|---|---|---|---|---|---|---|---|---|
| **Urine Sodium concentration (mmol/L)** | | | | | | | | | |
| <19 °C | F | 0.99 (0.93, 1.04) | 0.530 | 0.98 (0.92, 1.04) | 0.511 | 0.98 (0.92, 1.03) | 0.496 | 0.94 (0.90, 1.00) | 0.491 |
| | M | 1.01 (0.96, 1.07) | | 1.01 (0.96, 1.06) | | 1.01 (0.96, 1.06) | | 0.97 (0.92, 1.02) | |
| ≥19 °C | F | 1.14 (1.06, 1.22) | | 1.15 (1.06, 1.23) | | 1.15 (1.07, 1.23) | | 1.06 (1.00, 1.14) | |
| | M | 1.13 (1.06, 1.19) | | 1.13 (1.06, 1.19) | | 1.13 (1.06, 1.19) | | 1.04 (1.00, 1.09) | |
| **Urine Potassium concentration (mmol/L)** | | | | | | | | | |
| <16 °C | F | 0.93 (0.87, 0.99) | 0.181 | 0.92 (0.86, 0.98) | 0.156 | 0.92 (0.86, 0.98) | 0.138 | 0.87 (0.79, 0.97) | 0.099 |
| | M | 0.97 (0.91, 1.04) | | 0.97 (0.99, 1.04) | | 0.97 (0.99, 1.04) | | 0.93 (0.98, 1.06) | |
| ≥16 °C | F | 1.05 (1.01, 1.09) | | 1.05 (1.01, 1.09) | | 1.05 (1.01, 1.09) | | 1.03 (0.99, 1.07) | |
| | M | 1.08 (1.04, 1.13) | | 1.08 (1.04, 1.13) | | 1.08 (1.04, 1.13) | | 1.05 (1.01, 1.09) | |
| **Urine Magnesium concentration (mmol/L)** | | | | | | | | | |
| <16 °C | F | 1.11 (0.92, 1.34) | 0.300 | 1.11 (0.92, 1.34) | 0.311 | 1.09 (0.92, 1.32) | 0.377 | 1.06 (0.79, 1.42) | 0.340 |
| | M | 0.99 (0.83, 1.17) | | 0.99 (0.84, 1.17) | | 0.98 (0.83, 1.17) | | 0.95 (0.72, 1.25) | |
| ≥16 °C | F | 1.27 (1.17, 1.37) | | 1.27 (1.17, 1.38) | | 1.27 (1.17, 1.38) | | 0.93 (0.84, 1.03) | |
| | M | 1.30 (1.20, 1.40) | | 1.30 (1.20, 1.40) | | 1.30 (1.20, 1.40) | | 0.94 (0.83, 1.08) | |
| **Urine Calcium Concentration (mmol/L)** | | | | | | | | | |
| <20 °C | F | 1.38 (1.27, 1.49) | 0.891 | 1.36 (1.26, 1.48) | 0.969 | 1.36 (1.26, 1.49) | 0.968 | 1.15 (1.05, 1.27) | 0.839 |
| | M | 1.39 (1.23, 1.57) | | 1.37 (1.22, 1.57) | | 1.38 (1.22, 1.55) | | 1.18 (1.03, 1.35) | |
| ≥20 °C | F | 0.83 (0.75, 0.91) | | 0.82 (0.86, 1.12) | | 0.82 (0.86, 1.12) | | 0.93 (0.86, 1.01) | |
| | M | 0.81 (0.71, 0.92) | | 0.81 (0.71, 0.92) | | 0.81 (0.71, 0.92) | | 0.91 (0.81, 1.02) | |
| **Urine Chloride concentration (mmol/L)** | | | | | | | | | |
| <19 °C | F | 0.97 (0.92, 1.02) | 0.259 | 0.96 (0.91, 1.02) | 0.288 | 0.96 (0.91, 1.01) | 0.267 | 0.91 (0.87, 0.72) | 0.221 |
| | M | 1.01 (0.95, 1.07) | | 1.01 (0.95, 1.06) | | 1.00 (0.95, 1.06) | | 0.96 (0.91, 1.01) | |
| ≥19 °C | F | 1.15 (1.06, 1.23) | | 1.15 (1.07, 1.23) | | 1.15 (1.07, 1.23) | | 1.07 (1.00, 1.15) | |
| | M | 1.13 (1.07, 1.20) | | 1.13 (1.07, 1.20) | | 1.13 (1.07, 1.20) | | 1.05 (1.00, 1.09) | |

**Footnote**: Level of significance is 0.004 ($\alpha = 0.05/12$ or 0.004)

**TableS4**: Piecewise linear regression model showing associations between minimum temperature and 24-hour excretion of urine biomarkers for different minimum temperature segments using individual, household, and community level random intercepts. P-value indicates the overall significance of minimum temperature and sex interaction for all segments of ambient temperature using the Wald test.

| Temperature segments | Sex category | Model 1 | p-value | Model 2 | p-value | Model 3 | p-value | Model 4 | p-value |
|---|---|---|---|---|---|---|---|---|---|
| **Urine total protein excretion (mg/24 hour) $** | | | | | | | | | |
| <16 °C | F | 1.93 (1.40, 2.66) | 0.935 | 1.88 (1.38, 2.59) | 0.216 | 1.88 (1.38, 2.56) | 0.202 | 1.51 (1.13, 1.99) | 0.170 |
| | M | 1.93 (1.28, 2.94) | | 1.88(1.25, 2.80) | | 1.88 (1.26, 2.80) | | 1.48 (0.98, 2.25) | |
| ≥16 °C | F | 0.72 (0.66, 0.78) | | 0.71 (0.66, 0.76) | | 0.71 (0.66, 0.76) | | 0.91 (0.74, 1.11) | |
| | M | 0.72 (0.66, 0.78) | | 0.73 (0.68, 0.79) | | 0.73 (0.68, 0.79) | | 0.94 (0.79, 1.13) | |
| **Urine Magnesium excretion (mmol/24 hour) $** | | | | | | | | | |
| <16 °C | F | 1.17 (0.96, 1.43) | 0.525 | 1.16 (0.96, 1.42) | 0.239 | 1.15 (0.95, 1.39) | 0.297 | 1.09 (0.82, 1.48) | 0.304 |
| | M | 1.06 (0.90, 1.26) | | 1.03 (0.86, 1.22) | | 1.03 (0.86, 1.22) | | 0.98 (0.75, 1.27) | |
| ≥16°C | F | 1.12 (1.03, 1.21) | | 1.12 (1.04, 1.21) | | 1.12 (1.04, 1.21) | | 0.86 (0.77, 0.96) | |
| | M | 1.13 (1.05, 1.22) | | 1.15 (1.06, 1.23) | | 1.15 (1.06, 1.23) | | 0.88 (0.77, 1.00) | |
| **Urine Calcium excretion (mmol/24 hour) $** | | | | | | | | | |
| <20 °C | F | 1.34 (1.22, 1.45) | 0.744 | 1.32 (1.21, 1.45) | 0.999 | 1.32 (1.21, 1.46) | 0.999 | 1.15 (1.03, 1.28) | 0.926 |
| | M | 1.35 (1.19, 1.55) | | 1.32 (1.16, 1.51) | | 1.32 (1.17, 1.51) | | 1.17 (1.01, 1.36) | |
| ≥20 °C | F | 0.68 (0.61, 0.76) | | 0.68 (0.62, 0.76) | | 0.68 (0.62, 0.76) | | 0.84 (0.79, 0.90) | |
| | M | 0.66 (0.57, 0.76) | | 0.68 (0.59, 0.79) | | 0.68 (0.59, 0.79) | | 0.84 (0.76, 0.91) | |
| **Following variables weren't transformed to logarithm due to normal distribution** | | | | | | | | | |
| **Urine Sodium excretion (mmol/24 hour) ¥** | | | | | | | | | |
| <16 °C | F | -2.09 (-19.59, 15.41) | 0.210 | -2.75 (-22.00, 16.51) | 0.235 | -3.78 (-22.99, 15.44) | 0.269 | -2.08 (-18.05, 13.88) | 0.439 |
| | M | -7.55 (-29.79, 14.70) | | -10.69 (-31.83, 10.46) | | -11.06 (-32.53, 10.41) | | -6.71 (-26.34, 12.91) | |
| ≥16-<21 °C | F | -4.51 (-11.88, 2.86) | | -5.02 (-13.80, 3.76) | | -4.95 (-13.88, 3.98) | | -10.16 (-21.41, 1.09) | |
| | M | 3.25 (-5.47, 11.97) | | 2.97 (-5.95, 11.89) | | 3.01 (-5.81, 11.82) | | -3.89 (-16.09, 8.32) | |
| ≥21 °C | F | -6.26 (-15.34, 2.81) | | -5.13 (-14.87, 4.60) | | -4.99 (-14.72, 4.73) | | -5.39 (-12.55, 1.78) | |
| | M | -11. 35 (-21.00, -1.70) | | -7.34 (-17.06, 2.38) | | -7.20 (-16.91, 2.50) | | -6.21 (-14.66, 2.23) | |
| **Urine Potassium excretion (mmol/24 hour) ¥** | | | | | | | | | |
| <20 °C | F | -0.38 (-2.08, 1.32) | 0.131 | -0.58 (-2.43, 1.26) | 0.083 | -0.66 (-2.50, 1.18) | 0.065 | -1.28 (-3.07, 0.51) | 0.038 |
| | M | 1.25 (-0.12, 2.61) | | 0.85 (-0.66, 2.36) | | 0.84 (-0.67, 2.35) | | 0.47 (-1.47, 2.40) | |
| ≥20 °C | F | -3.84 (-5.56, -2.12) | | -3.59 (-5.53, -1.65) | | -3.54 (-5.48, -1.59) | | -1.90 (-3.59, -0.21) | |
| | M | -3.98 (-6.32, -1.64) | | -2.98 (-5.50, -0.46) | | -2.94 (-5.46, -0.41) | | -1.51 (-3.11, 0.09) | |
| **Urine Chloride excretion (mmol/24 hour) ¥** | | | | | | | | | |
| <16 °C | F | -8.11 (-27.92, 11.71) | 0.009 | -9.53 (-31.55, 12.49) | 0.029 | -10.65 (-32.65, 11.35) | 0.039 | -9.98 (-25.69, 5.73) | 0.074 |
| | M | -14.82 (-38.13, 8.49) | | -18.30 (-40.15, 3.55) | | -18.59, (-40.87, 3.68) | | -14.49 (-33.54, 4.56) | |
| ≥16-<21 °C | F | -5.28 (-13.99, 3.43) | | -5.39 (-15.65, 4.86) | | -5.29 (-15.70, 5.11) | | -13.28 (-24.94, -1.61) | |
| | M | 7.48 (-2.13, 17.09) | | 7.26 (-2.83, 17.35) | | 7.30 (-2.66, 17.25) | | -2.04 (-15.22, 11.14) | |
| ≥21 °C | F | -6.00 (-17.04, 5.03) | | -4.76 (-16.17, 6.65) | | -4.64 (-16.02, 6.75) | | -2.43 (-12.35, 7.49) | |
| | M | -14.41 (-26.09, -2.73) | | -10.36 (-22.58, 1.85) | | -10.23 (-22.31, 1.86) | | -7.05 (-15.22, 1.12) | |
| **Urine volume (liter/24 hour) ¥** | | | | | | | | | |
| <16 °C | F | 0.17 (0.09, 0.26) | 0.526 | 0.18 (0.09, 0.27) | 0.385 | 0.18 (0.09, 0.27) | 0.392 | 0.16 (-0.02, 0.33) | 0.429 |
| | M | 0.07 (-0.14, 0.28) | | 0.05 (-0.16, 0.27) | | 0.05 (-0.17, 0.27) | | 0.05 (-0.20, 0.30) | |
| ≥16 °C | F | -0.25 (-0.28, -0.22) | | -0.25 (-0.28, -0.22) | | -0.25 (-0.28, -0.22) | | -0.13 (-0.19, -0.07) | |
| | M | -0.24 (-0.28, -0.20) | | -0.24 (-0.28, -0.20) | | -0.24 (-0.28, -0.20) | | -0.11 (-0.18, -0.04) | |

**Footnote**: Level of significance is 0.004 ($\alpha = 0.05/12$ or 0.004)

$ Regression co-efficient and 95% confidence intervals represent geometric mean ratio in relation to 5℃ increases in minimum ambient temperature.

¥ Indicate change in mean biomarkers due to 5℃ increases in minimum ambient temperature.

**Table S5:** Piecewise linear quantile model showing association between daily average temperature and the concentration of urine biomarkers for different average temperature segments using individual level random intercepts. P-value indicates the overall significance of average temperature and sex interaction for all segments of ambient temperature using the Wald test.

| Temperature segments | Sex category | Model 1 | p-value | Model 2 | p-value | Model 3 | p-value | Model 4 | p-value |
|---|---|---|---|---|---|---|---|---|---|
| **Urine Sodium concentration (mmol/L)** | | | | | | | | | |
| <24 °C | F | 1.51 (-4.34, 7.35) | 0.850 | 0.81 (-3.26, 4.87) | 0.679 | 0.84 (-3.34, 5.02) | 0.728 | -4.48 (-12.26, 3.29) | 0.821 |
| | M | 4.26 (-3.59, 12.10) | | 3.73 (-1.77, 9.22) | | 3.44 (-2.21, 9.08) | | -1.81 (-11.25, 7.63) | |
| ≥24 °C | F | 21.16 (14.48, 27.85) | | 21.86 (17.02, 26.70) | | 21.86 (17.03, 26.70) | | 12.49 (2.11, 22.88) | |
| | M | 18.65 (9.92, 27.38) | | 18.96 (12.76, 25.16) | | 19.00 (12.63, 25.37) | | 9.03 (-2.98, 21.04) | |
| **Urine Potassium concentration (mmol/L)** | | | | | | | | | |
| <21 °C | F | -1.14 (-11.25, 8.96) | 0.962 | -1.18 (-4.13, 1.77) | 0.321 | -1.18 (-3.09, 0.72) | 0.051 | -1.60 (-3.66, 0.45) | 0.037 |
| | M | 0.79 (-14.00, 15.58) | | 0.75 (-3.58, 5.08) | | 0.60 (-2.17, 3.38) | | 0.87 (-1.93, 3.67) | |
| ≥21-<26 °C | F | 2.66 (-3.47, 8.79) | | 2.63 (0.84, 4.43) | | 2.65 (1.49, 3.80) | | 0.60 (-0.85, 2.05) | |
| | M | 3.64 (-4.96, 12.24) | | 3.52 (1.02, 6.01) | | 3.50 (1.91, 5.10) | | 1.18 (-0.67, 3.02) | |
| ≥26 °C | F | 0.36 (-9.63, 10.36) | | 0.48 (-2.46, 3.42) | | 0.47 (-1.43, 2.36) | | 1.89 (-0.34, 4.12) | |
| | M | 1.13 (-11.96, 14.21) | | 1.51 (-2.29, 5.32) | | 1.50 (-0.95, 3.94) | | 2.99 (0.27, 5.72) | |
| **Urine Magnesium concentration (mmol/L)** | | | | | | | | | |
| <21 °C | F | 0.25 (-0.56, 1.05) | 0.968 | 0.26 (-0.40, 0.92) | 0.949 | 0.27 (-0.30, 0.84) | 0.929 | 0.23 (-0.75, 1.20) | 0.988 |
| | M | 0.03 (-1.11, 1.17) | | -0.03 (-0.90, 0.96) | | 0.02 (-0.79, 0.83) | | 0.03 (-1.26, 1.33) | |
| ≥21-<25 °C | F | 0.29 (-0.36, 0.93) | | 0.28 (-0.25, 0.81) | | 0.28 (-0.18, 0.74) | | -0.18 (-1.03, 0.67) | |
| | M | 0.52 (-0.32, 1.37) | | 0.50 (-0.19, 1.19) | | 0.49 (-0.11, 1.09) | | 0.01 (-1.02, 1.05) | |
| ≥25°C | F | 1.18 (0.45, 1.91) | | 1.20 (0.59, 1.81) | | 1.20 (0.67, 1.73) | | 0.06 (-1.02, 1.14) | |
| | M | 1.13 (0.22, 2.04) | | 1.17 (0.42, 1,93) | | 1.18 (0.52, 1.84) | | -0.01 (-1.23, 1.23) | |
| **Urine Calcium Concentration (mmol/L)** | | | | | | | | | |
| <21 °C | F | -0.24 (-0.48, 0.01) | 0.763 | -0.23 (-0.48, 0.01) | 0.640 | -0.23 (-0.47, 0.01) | 0.629 | 0.18 (-0.10, 0.46) | 0.679 |
| | M | -0.17 (-0.52, 0.18) | | -0.19 (-0.54, 0.15) | | -0.19 (-0.54, 0.15) | | 0.28 (-0.11, 0.68) | |
| ≥21-<25 °C | F | 1.72 (1.53, 1.91) | | 1.73 (1.54, 1.91) | | 1.73 (1.54, 1.92) | | 0.71 (0.47, 0.95) | |
| | M | 1.57 (1.33, 1.81) | | 1.55 (1.31, 1.78) | | 1.55 (1.31, 1.78) | | 0.51 (0.22, 0.80) | |
| ≥25 °C | F | -1.16 (-1.34, -0.98) | | -1.18 (-1.36, -1.00) | | -1.18 (-1.36, -1.00) | | -0.30 (-0.55, -0.05) | |
| | M | -1.09 (-1.31, -0.87) | | -1.05(-1.27, -0.84) | | -1.05 (-1.27, -0.84) | | -0.14 (-0.43, 0.15) | |
| **Urine Chloride concentration (mmol/L)** | | | | | | | | | |
| <24 °C | F | 0.70 (-34.09, 35.49) | 0.989 | 0.19 (-3.38, 4.20) | 0.450 | 0.19 (-4.05, 4.43) | 0.542 | -9.57 (-17.12, -2.03) | 0.705 |
| | M | 4.97 (-43.01, 52.95) | | 4.46 (-1.07, 9.99) | | 4.20 (-1.62, 10.03) | | -5.48 (-14.66, 3.71) | |
| ≥24 °C | F | 21.89 (-16.75, 60.53) | | 22.58 (18.06, 27.10) | | 22.68 (17.91, 27.45) | | 15.32 (5.61, 25.02) | |
| | M | 20.35 (-31.78, 72.48) | | 20.68 (14.57, 26.78) | | 20.60 (14.19, 27.02) | | 12.63 (1.17, 24.09) | |

**Footnote:** Level of significance is 0.004 ($\alpha = 0.05/12$ or 0.004).

**Table S6:** Piecewise linear quantile model showing association between daily average temperature and the 24-hour urine biomarkers for different average temperature segments using individual level random intercepts. P-value indicates the overall significance of average temperature and sex interaction for all segments of ambient temperature using the Wald test.

| Temperature segments | Sex category | Model 1 | p-value | Model 2 | p-value | Model 3 | p-value | Model 4 | p-value |
|---|---|---|---|---|---|---|---|---|---|
| **Urine Sodium excretion (mmol/24 hour)** | | | | | | | | | |
| <21 °C | F | 6.48 (-6.08, 19.05) | 0.986 | 2.68 (-11.42, 16.79) | 0.969 | 2.29 (-12.03, 16.60) | 0.981 | 6.04 (-8.04, 20.12) | 0.970 |
| | M | 8.16 (-7.34, 23.65) | | -3.97 (-8.40, 0.46) | | 1.29 (-16.77, 19.35) | | 6.02 (-11.61, 23.66) | |
| ≥21 °C | F | -5.04 (-8.40, -1.68) | | -4.58 (-7.95, -1.21) | | -3.88 (-8.36, 0.60) | | -6.70 (-12.81, -0.58) | |
| | M | -5.36 (-9.67, -1.05) | | -3.24 (-8.74, 2.26) | | -3.29 (-8.89, 2.30) | | -6.10 (-12.86, 0.66) | |
| **Urine Potassium excretion (mmol/24 hour)** | | | | | | | | | |
| <25 °C | F | 1.44 (0.27, 2.61) | 0.032 | 1.31 (0.11, 2.50) | 0.023 | 1.31 (0.11, 2.50) | 0.023 | 0.55 (-1.09, 2.20) | 0.016 |
| | M | 3.20 (1.92, 4.49) | | 2.74 (1.42, 4.07) | | 2.74 (1.42, 4.06) | | 2.03 (0.24, 3.83) | |
| ≥25 °C | F | -7.09 (-8.70, -5.48) | | -6.64 (-8.54, -4.75) | | -6.65 (-8.52, -4.77) | | -2.52 (-4.82, -0.23) | |
| | M | -7.06 (-8.99, -5.11) | | -5.82(-8.14, -3.50) | | -5.82 (-8.14, -3.51) | | -1.59 (-4.25, 1.08) | |
| **Urine Magnesium excretion (mmol/24 hour)** | | | | | | | | | |
| <21 °C | F | 0.73 (-2.19, 3.64) | 0.988 | 0.71 (0.09, 1.34) | 0.612 | 0.73 (-0.16, 1.63) | 0.778 | 1.15 (-3.85, 6.16) | 0.993 |
| | M | 0.42 (-3.36, 4.21) | | 0.30 (-0.51, 1.26) | | 0.29 (-0.87, 1.45) | | 0.75 (-5.43, 6.92) | |
| ≥21°C | F | 0.59 (-0.34, 1.53) | | 0.60 (0.40, 0.81) | | 0.60 (0.31, 0.90) | | -0.72 (-3.23, 1.79) | |
| | M | 0.70 (-0.47, 1.87) | | 0.75 (0.49, 1.00) | | 0.75 (0.38, 1.11) | | -0.60 (-3.35, 2.14) | |
| **Urine Calcium excretion (mmol/24 hour)** | | | | | | | | | |
| <21 °C | F | 0.02 (-0.57, 0.61) | 0.812 | -0.05 (-0.64, 0.54) | 0.731 | -0.03 (-0.61, 0.56) | 0.740 | 0.98 (0.35, 1.62) | 0.784 |
| | M | -0.05 (-0.82, 0.71) | | -0.24 (-0.10, 0.52) | | -0.22 (-0.97, 0.53) | | 0.98 (0.17, 1.79) | |
| ≥21-<25 °C | F | 3.06 (2.64, 3.49) | | 3.09 (2.67, 3.51) | | 3.08 (2.67, 3.50) | | 1.23 (0.72, 1.75) | |
| | M | 2.91 (2.39, 3.43) | | 2.87 (2.36, 3.38) | | 2.87 (2.36, 3.37) | | 0.99 (0.39, 1.59) | |
| ≥25 °C | F | -2.95 (-3.34, -2.57) | | -2.97 (-3.36, -2.58) | | -2.96 (-3.34, -2.58) | | -1.27 (-1.79, -0.73) | |
| | M | -2.98 (-3.45, -2.51) | | -2.76 (-3.24, -2.29) | | -2.76 (-3.24, -2.29) | | -0.96 (-1.56, -0.35) | |
| **Urine Chloride excretion (mmol/24 hour)** | | | | | | | | | |
| <21 °C | F | -6.23 (-22. 12, 9.66) | 0.793 | -9.80 (-39.57, 19.98) | 0.974 | -10.33 (-31.43, 10.77) | 0.938 | -3.70 (-21.13, 13.72) | 0.913 |
| | M | -8.60 (-27.27, 10.06) | | -13.98 (-48.61, 20.66) | | -13.71 (-38.27, 10.86) | | -2.96 (-23.51, 17.58) | |
| ≥21-<25 °C | F | 2.91 (-7.45, 13.27) | | 3.69 (-15.57, 22.94) | | 3.89 (-9.75, 17.52) | | -14.53 (-27.49, -1.58) | |
| | M | 10.57 (-2.12, 23.25) | | -9.87 (-12.71, 32.65) | | 9.97 (-6.07, 26.02) | | -10.88 (-25.99, 4.23) | |
| ≥25 °C | F | -11.81 (-20.67, -2.95) | | -9.87 (-27.71, 7.96) | | -9.82 (-22.44, 2.81) | | 2.90 (-9.79, 15.59) | |
| | M | -16.69 (-27.69, -5.70) | | -12.05 (-34.52, 10.43) | | -12.26 (-28.17 3.66) | | 2.33 (-12.44, 17.11) | |
| **Urine total protein excretion (mg/24 hour)** | | | | | | | | | |
| <23°C | F | -6.81 (-588.94, 575.33) | 0.979 | 33.93 (-204.61, 272.49) | 0.972 | 33.38 (-116.52 183.28) | 0.935 | -8.46 (-490.44, 473.52) | 0.992 |
| | M | 4.86 (-463.34, 473.06) | | -3.82 (-269.11, 261.47) | | -3.07 (-169.91, 163.77) | | -44.43 (-610.72, 521.86) | |
| ≥23 °C | F | 1.92 (-2.32, 6.17) | | -94.22 (-277.20, 88. 76) | | -94.26 (-209.02, 20.51) | | -56.28 (-358.36, 245.80) | |
| | M | -99.38 (-419.78, 221.03) | | -93.86 (-278.89, 91.17) | | -93.77 (-209.91, 22.37) | | -54.99 (-521.73, 411.75) | |
| **Urine volume (liter/24 hour)** | | | | | | | | | |
| <22 °C | F | 0.20 (0.11, 0.30) | 0.899 | 0.20 (0.10, 0.29) | 0.833 | 0.20 (0.10, 0.29) | 0.866 | 0.30 (0.18, 0.43) | 0.999 |
| | M | 0.19 (0.08, 0.31) | | 0.18 (0.07, 0.30) | | 0.19 (0.07, 0.31) | | 0.30 (0.16, 0.45) | |
| ≥22 °C | F | -0.36 (-0.40, -0.32) | | -0.36 (-0.40, -0.32) | | -0.36 (-0.40, -0.32) | | -0.21 (-0.29, -0.14) | |
| | M | -0.37 (-0.42, -0.32) | | -0.37 (-0.42, -0.32) | | -0.37 (-0.43, -0.32) | | -0.21 (-0.30, -0.13) | |

**Footnote:** Level of significance is 0.004 ($\alpha = 0.05/12$ or 0.004).

## Supplementary figures:

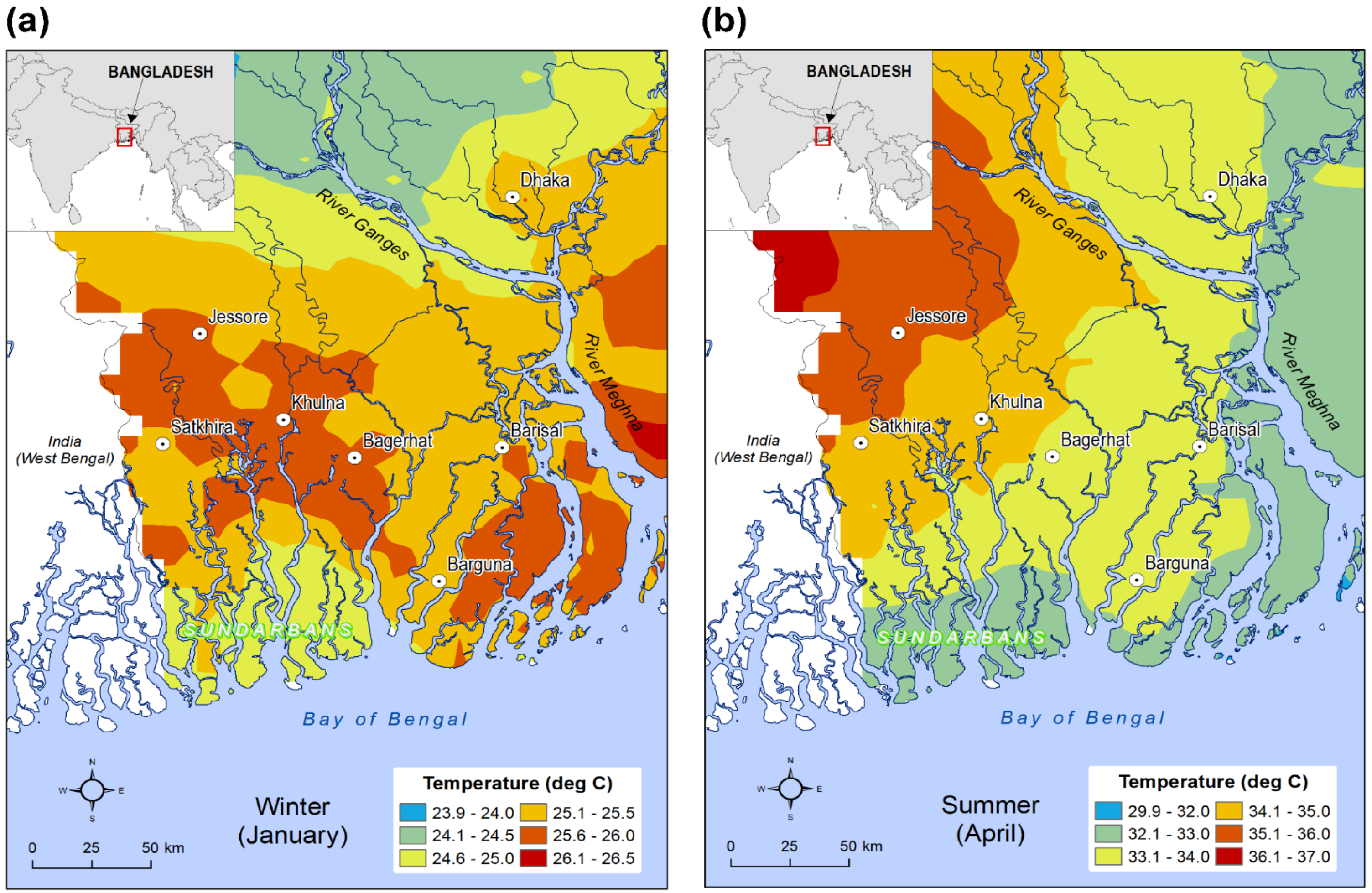


**Figure S1:** Map of the study area (Khulna, Satkhira, and Bagerhat) in Southwest coastal Bangladesh.

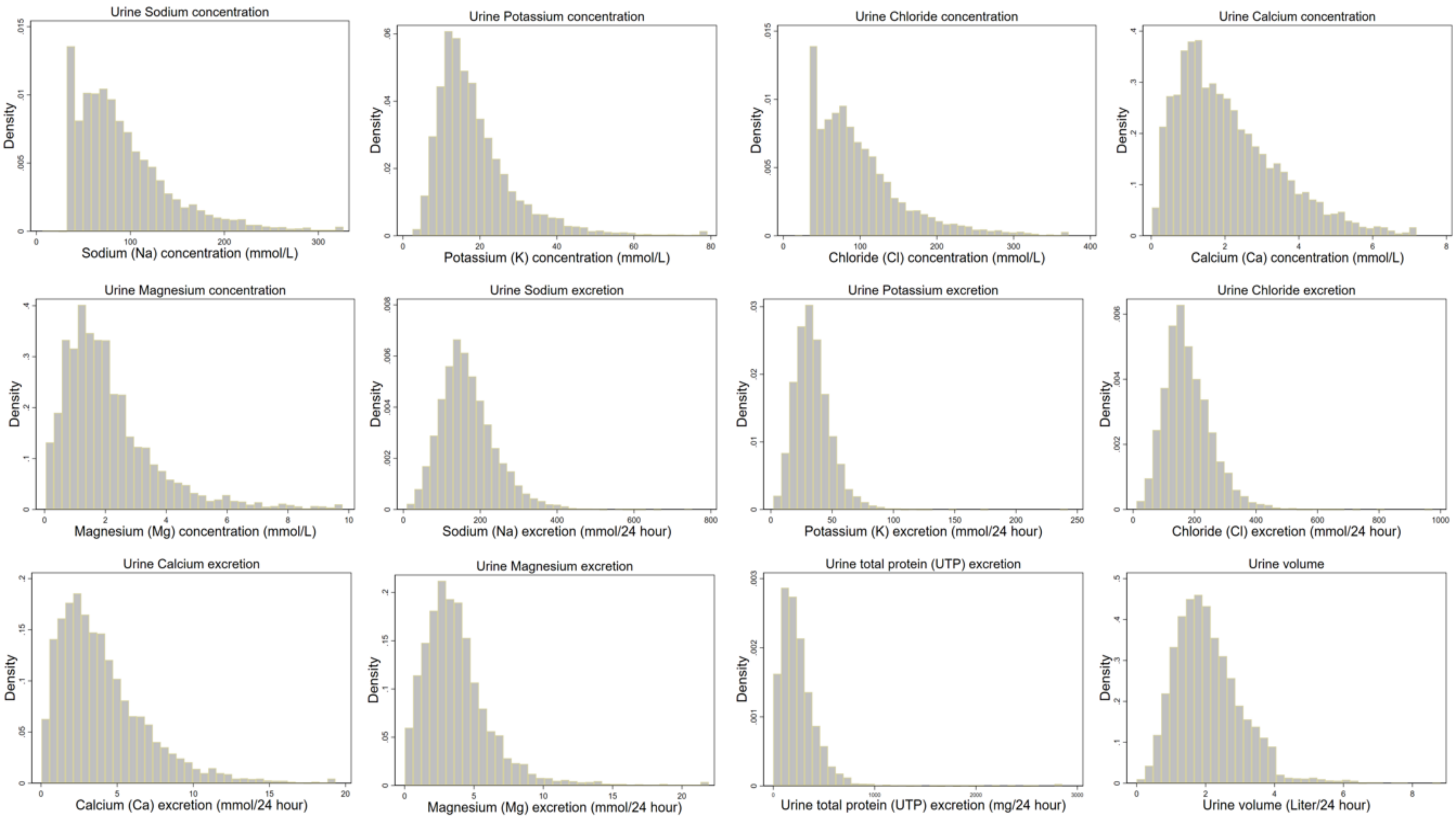


**Figure S2:** Histogram of the concentration and 24-hour excretion of urinary electrolytes (Sodium, Potassium, Chloride, Calcium, and Magnesium) including 24-hour excretion of urine total protein and volume.

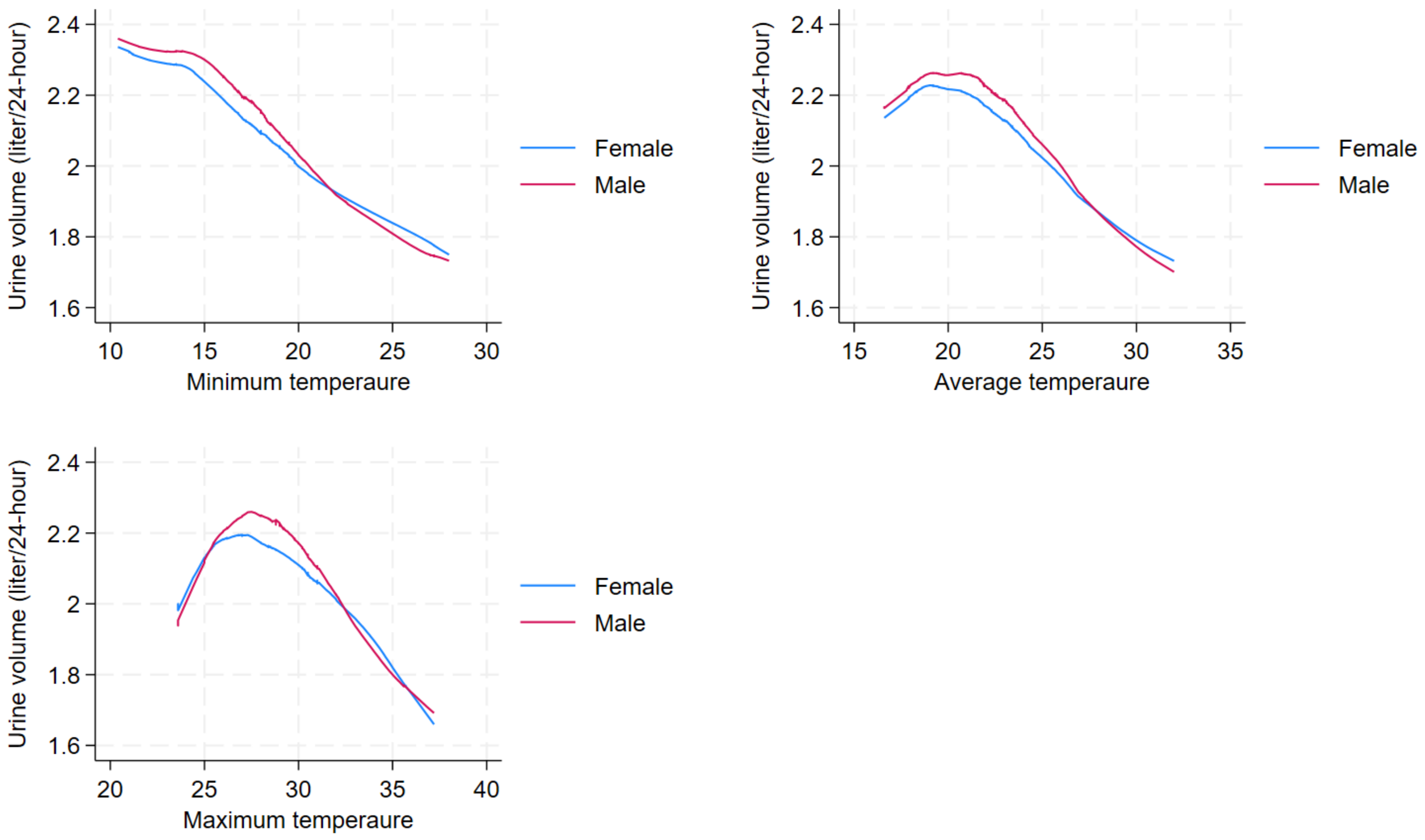


**Figure S3**: Relationship between ambient temperature (minimum, average, and maximum) and 24-hour urine volume for male and female.

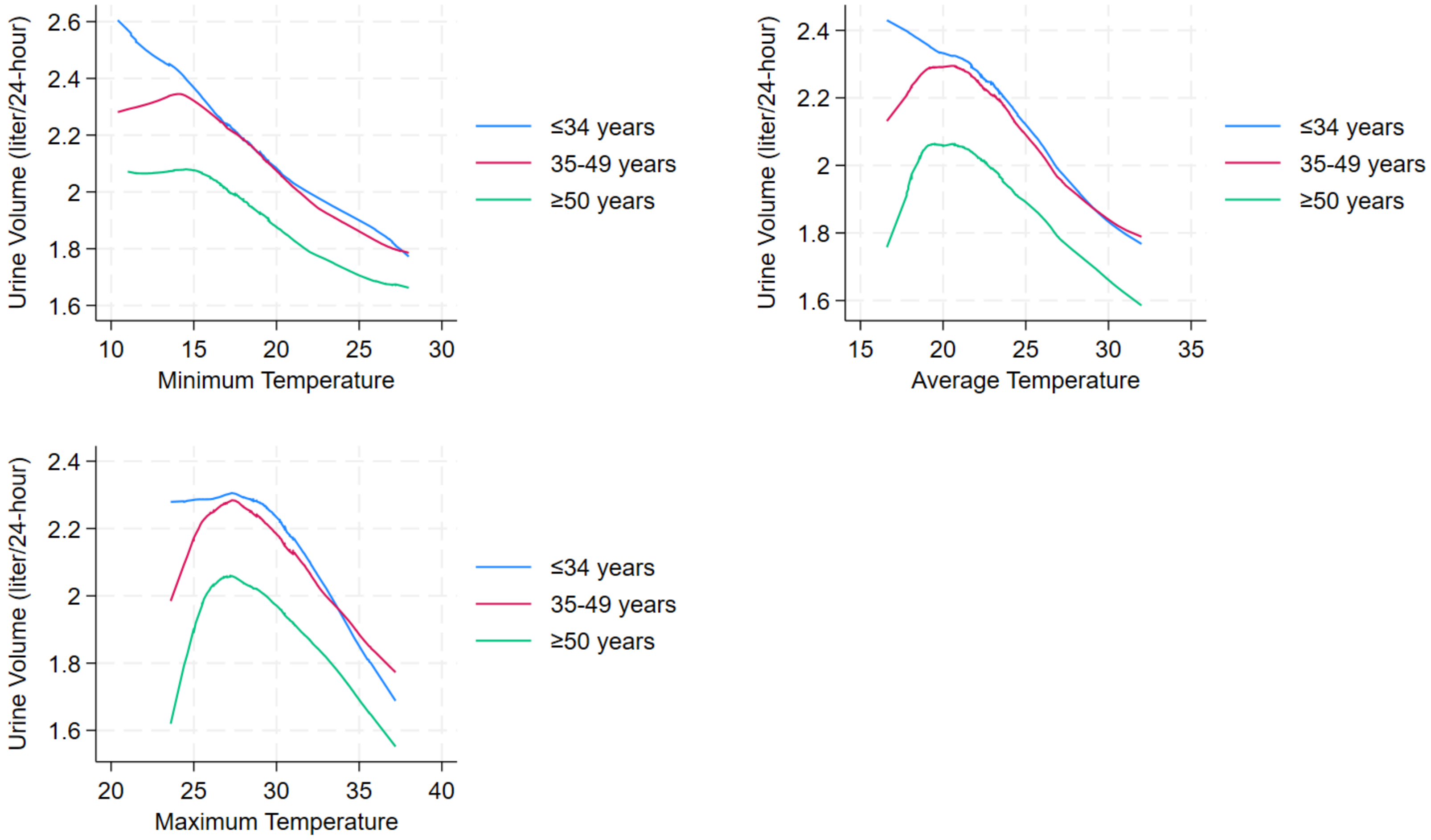


**Figure S4**: Relationship between ambient temperature (minimum, average, and maximum) and 24-hour urine volume for different age-groups.

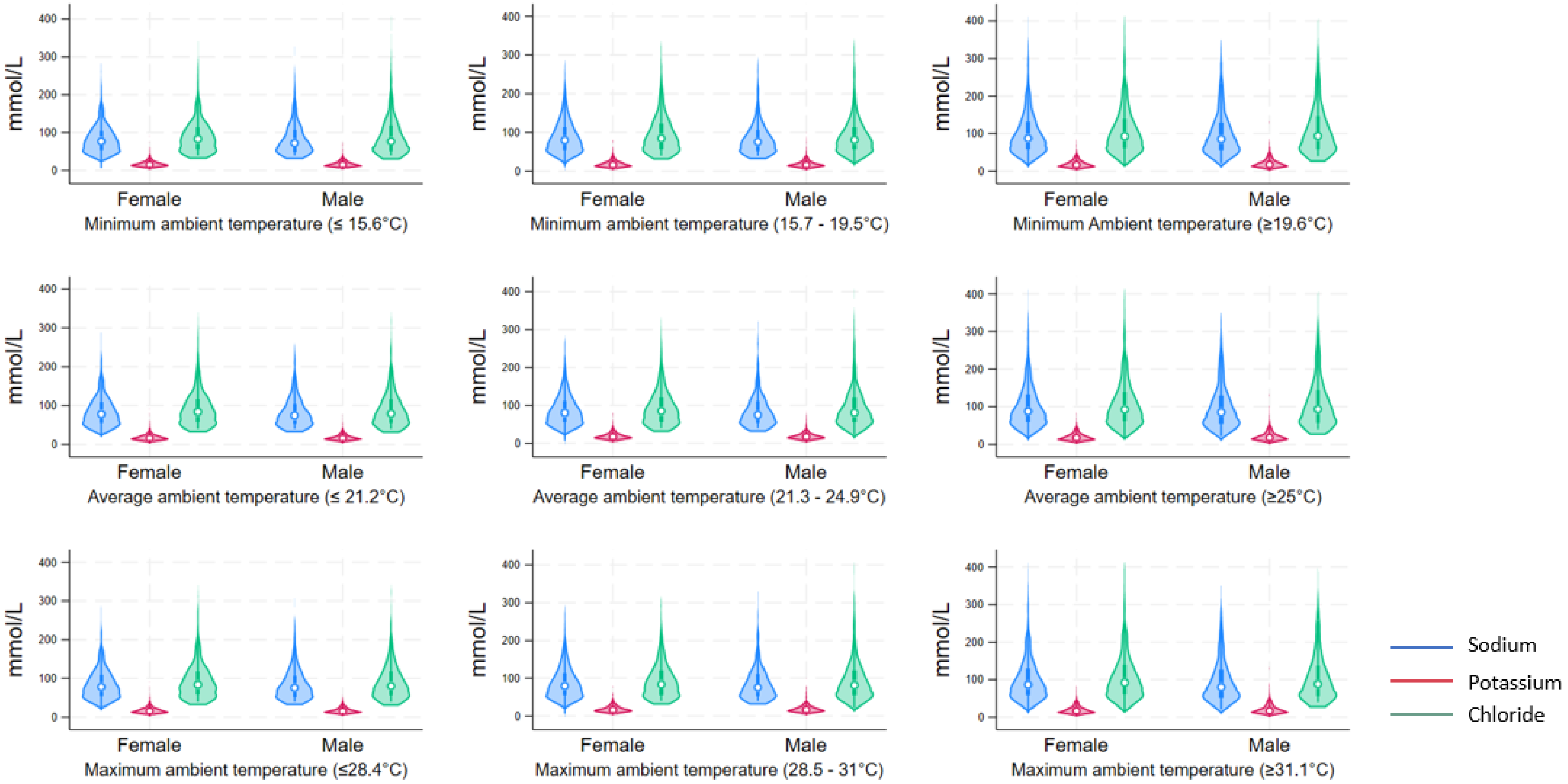


**Figure S5**: Violin plots illustrating the concentration of urine electrolytes (sodium, potassium, and chloride) for male and female across tertiles of ambient temperature (Minimum ambient temperature: tertile 1 : ≤ 15.6 ◦C, tertile 2: 15.7 – 19.5◦C, tertile 3: ≥ 19.6 ◦C; Average ambient temperature: tertile 1 : ≤ 21.2 ◦C, tertile 2: 21.3 – 24.9◦C, tertile 3: ≥ 25 ◦C; Maximum ambient temperature: tertile 1 : ≤ 28.4 ◦C, tertile 2: 28.5 – 31◦C, tertile 3: ≥ 31.1 ◦C).

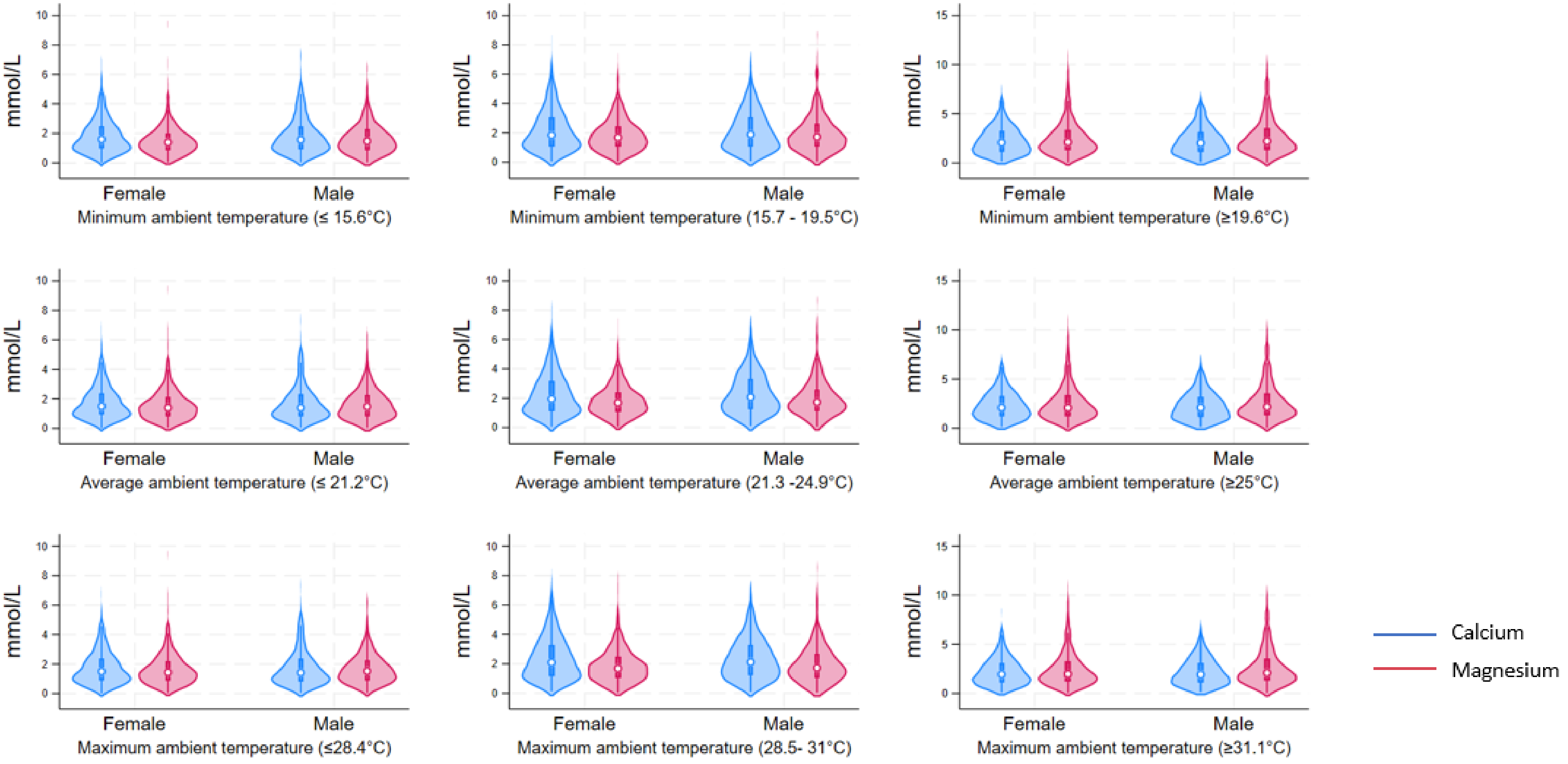


**Figure S6**: Violin plots illustrating the concentration of urine electrolytes (calcium and magnesium) for male and female across tertiles of ambient temperature (Minimum ambient temperature: tertile 1 : ≤ 15.6 ◦C, tertile 2: 15.7 – 19.5◦C, tertile 3: ≥ 19.6 ◦C; Average ambient temperature: tertile 1 : ≤ 21.2 ◦C, tertile 2: 21.3 – 24.9◦C, tertile 3: ≥ 25 ◦C; Maximum ambient temperature: tertile 1 : ≤ 28.4 ◦C, tertile 2: 28.5 – 31◦C, tertile 3: ≥ 31.1 ◦C).

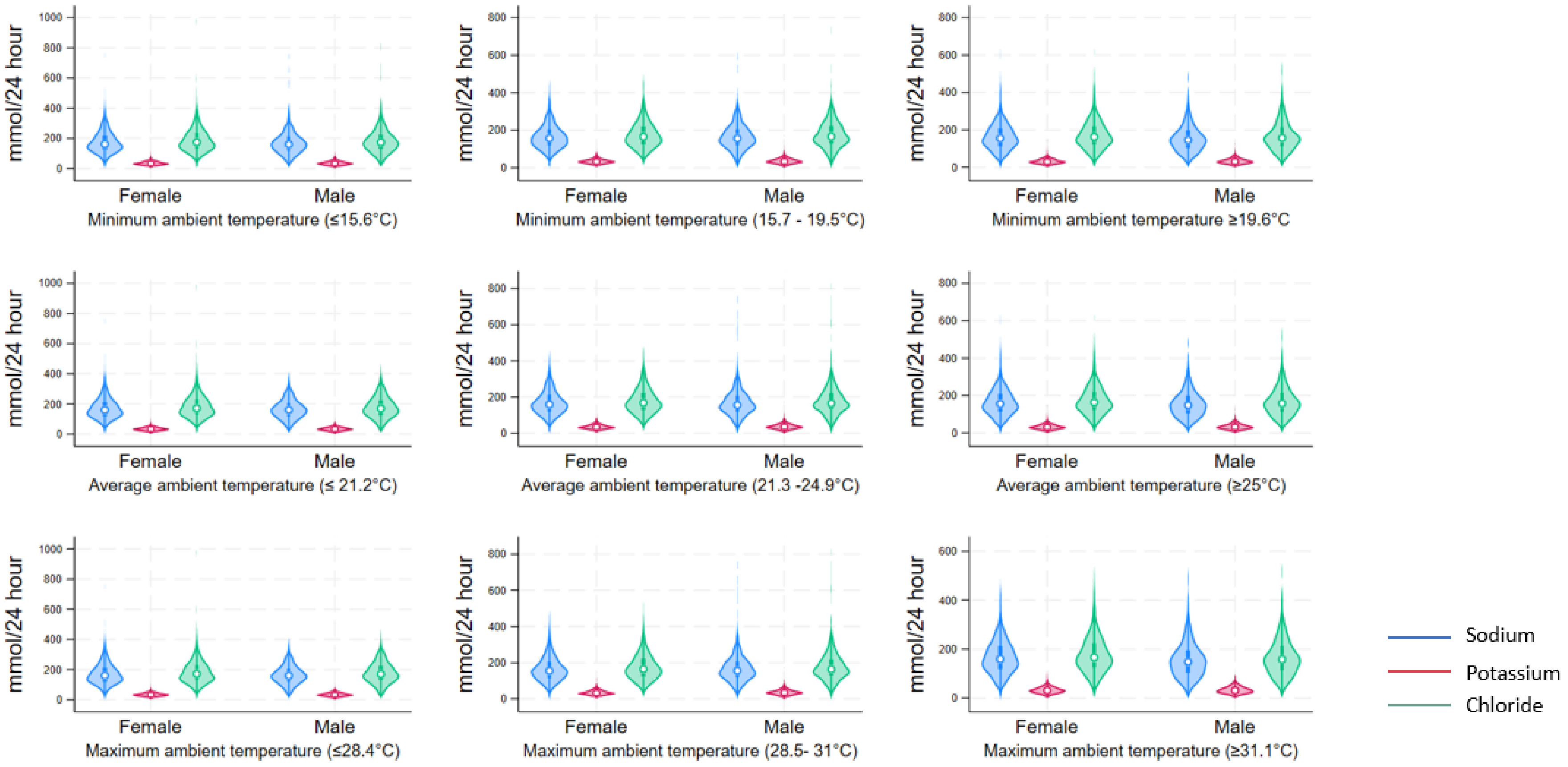


**Figure S7**: Violin plots illustrating 24-h excretion of urine electrolytes (sodium, potassium, and chloride) for male and female across tertiles of ambient temperature (Minimum ambient temperature: tertile 1 : ≤ 15.6 ◦C, tertile 2: 15.7 – 19.5◦C, tertile 3: ≥ 19.6 ◦C; Average ambient temperature: tertile 1 : ≤ 21.2 ◦C, tertile 2: 21.3 – 24.9◦C, tertile 3: ≥ 25 ◦C; Maximum ambient temperature: tertile 1 : ≤ 28.4 ◦C, tertile 2: 28.5 – 31◦C, tertile 3: ≥ 31.1 ◦C).

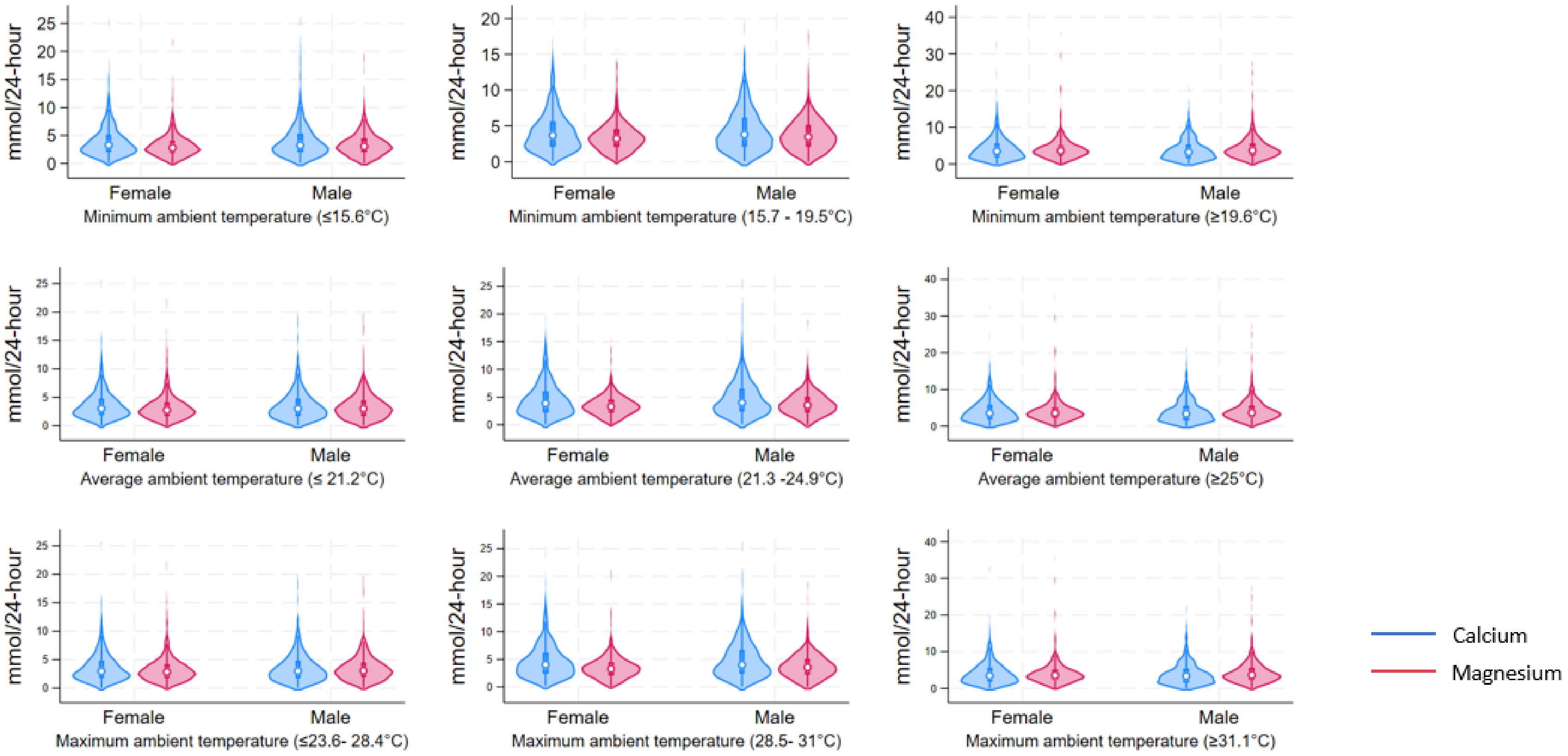


**Figure S8**: Violin plots illustrating 24-h excretion of urine electrolytes (calcium and magnesium) for male and female across tertiles of ambient temperature (Minimum ambient temperature: tertile 1 : ≤ 15.6 ◦C, tertile 2: 15.7 – 19.5◦C, tertile 3: ≥ 19.6 ◦C; Average ambient temperature: tertile 1 : ≤ 21.2 ◦C, tertile 2: 21.3 – 24.9◦C, tertile 3: ≥ 25 ◦C; Maximum ambient temperature: tertile 1 : ≤ 28.4 ◦C, tertile 2: 28.5 – 31◦C, tertile 3: ≥ 31.1 ◦C).

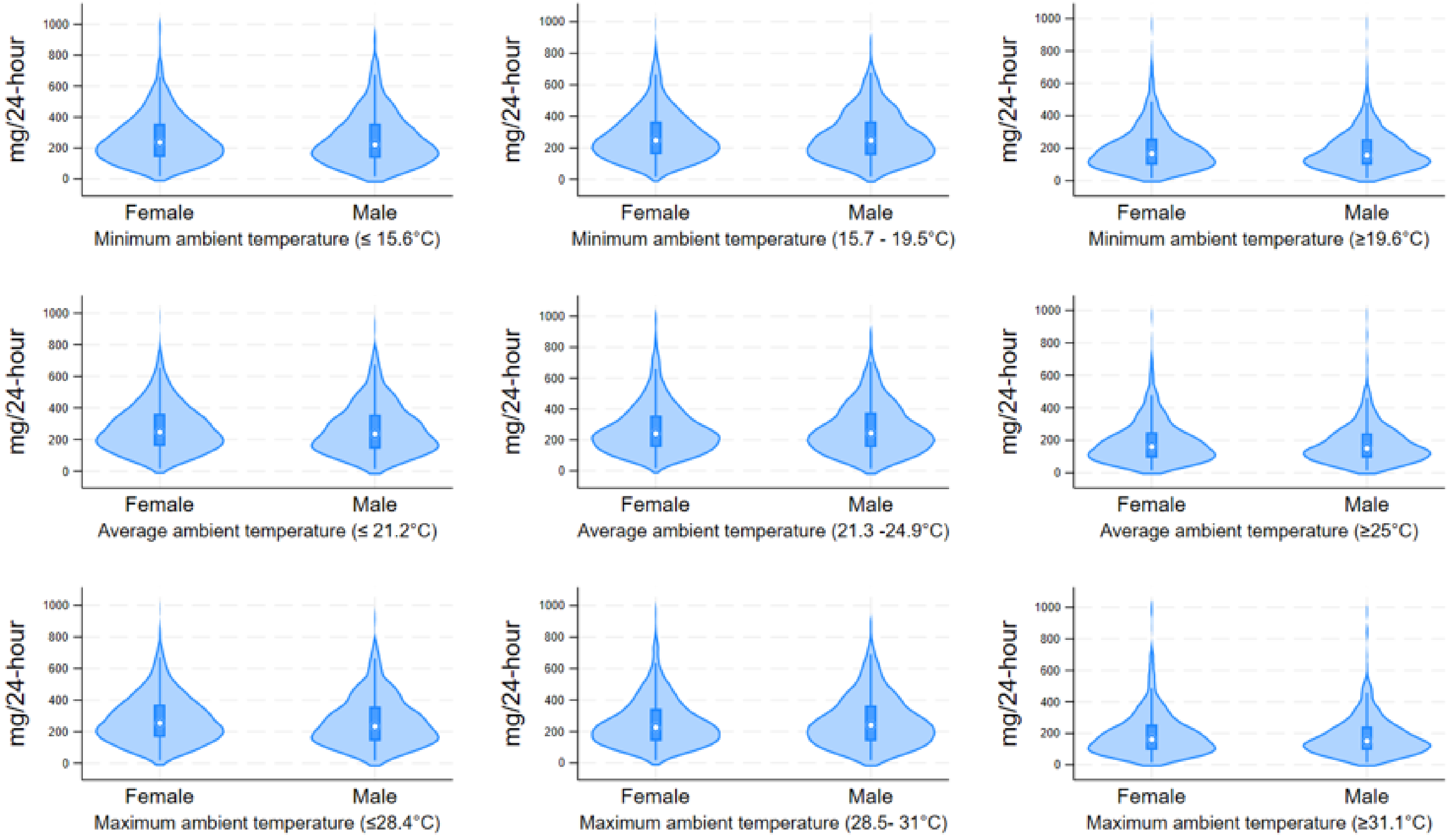


**Figure S9**: Violin plots illustrating 24-h excretion of urine total protein (UTP) for male and female across tertiles of ambient temperature (Minimum ambient temperature:  tertile 1 : ≤ 15.6 ◦C, tertile 2: 15.7 – 19.5◦C, tertile 3: ≥ 19.6 ◦C; Average ambient temperature: tertile 1 : ≤ 21.2 ◦C, tertile 2: 21.3 – 24.9◦C, tertile 3: ≥ 25 ◦C; Maximum ambient temperature: tertile 1 : ≤ 28.4 ◦C, tertile 2: 28.5 – 31◦C, tertile 3: ≥ 31.1 ◦C).

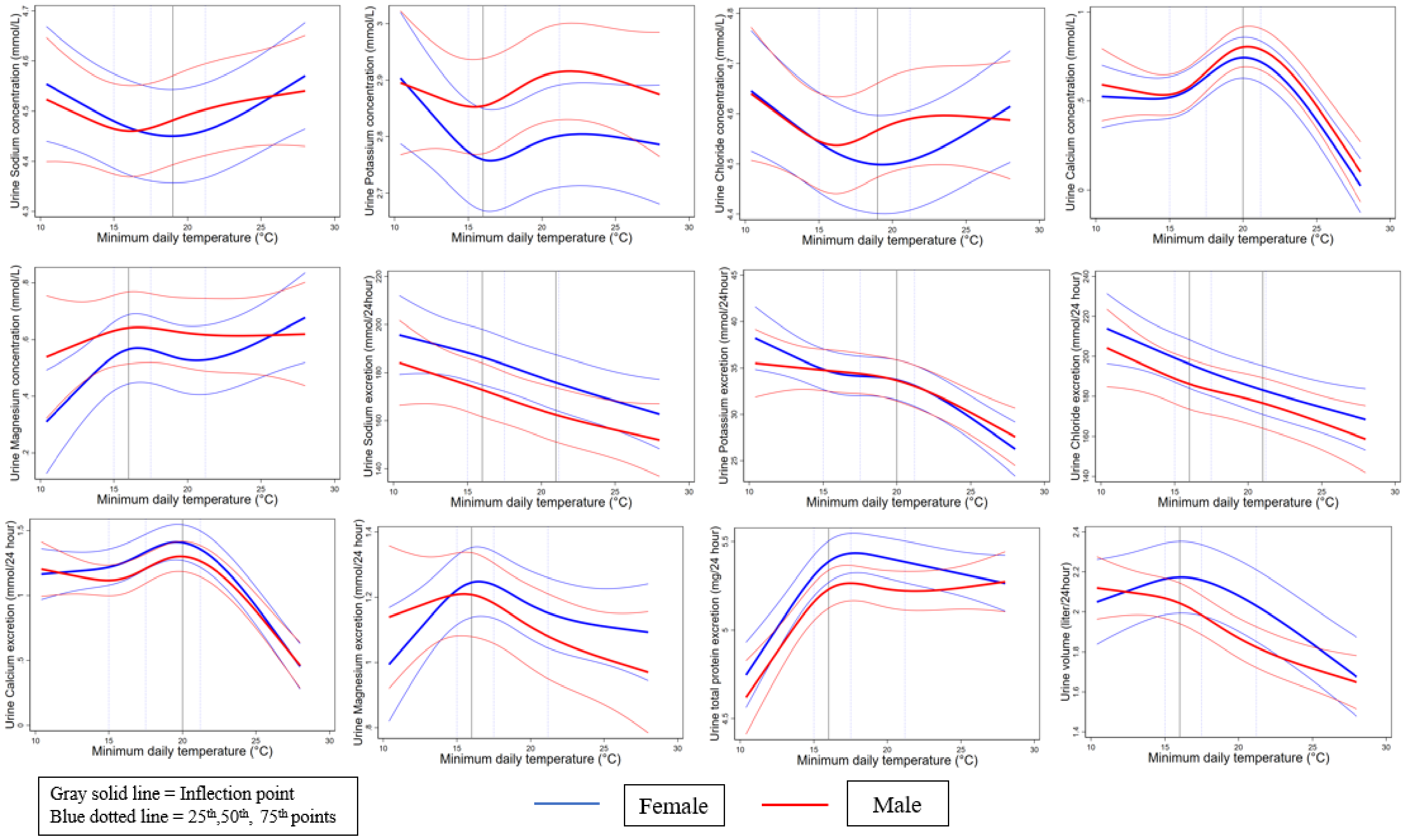


**Figure S10:** Restricted cubic spline plots with their 95% confidence bands using linear mixed-effect model demonstrating sex-stratified association between the concentration and 24-hour excretion of urinary electrolytes (Sodium, Potassium, Chloride, Calcium, and Magnesium) and 24-hour excretion of urine total protein and volume with **minimum ambient temperature**, adjusted for age, BMI, physical exercise, smoking, alcohol consumption, sleep duration, religion, household wealth, time of visit, drinking water salinity, and humidity.

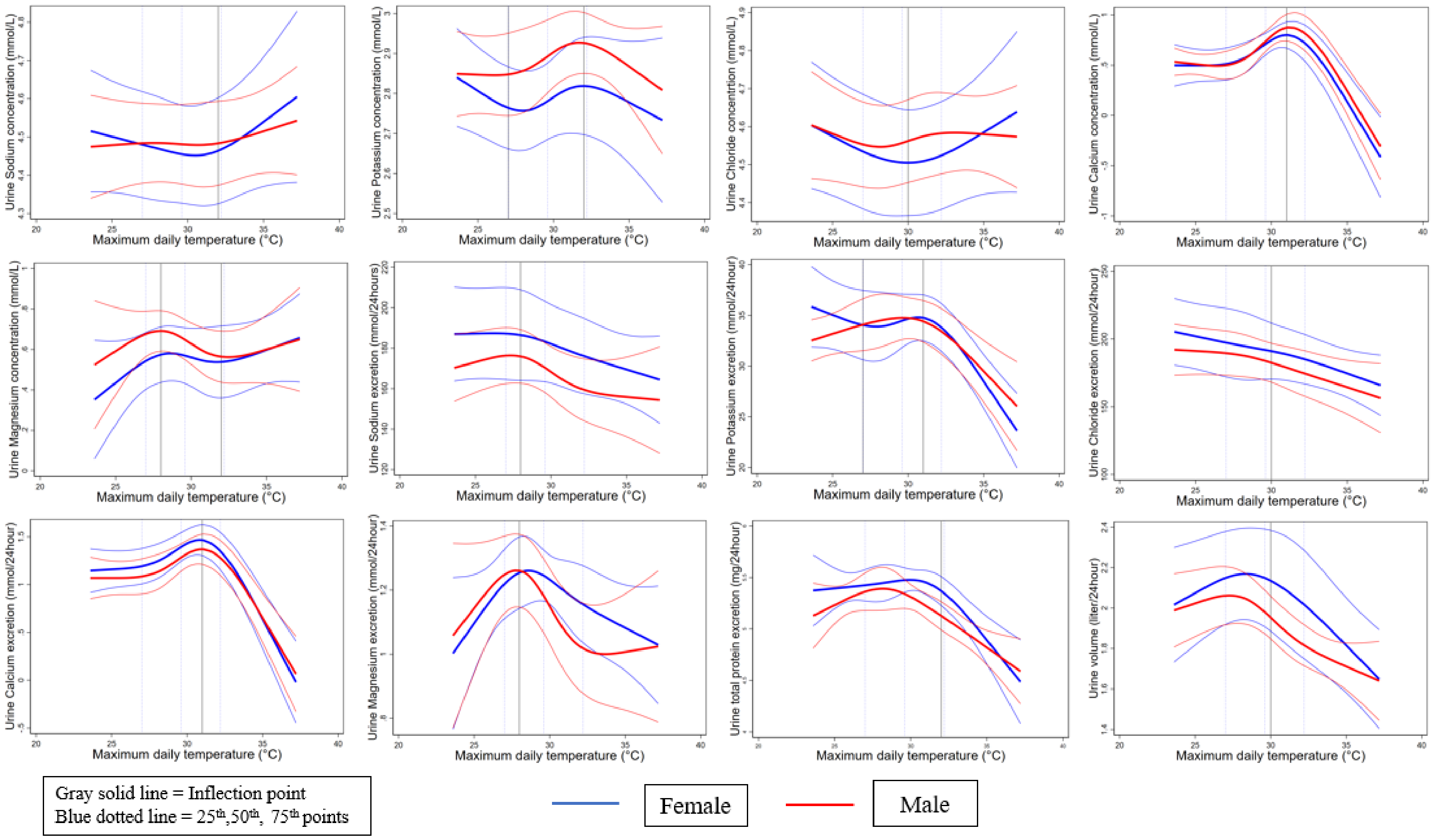


**Figure S11:** Restricted cubic spline plots with their 95% confidence bands using linear mixed-effect model demonstrating sex-stratified association between the concentration and 24-hour excretion of urinary electrolytes (Sodium, Potassium, Chloride, Calcium, and Magnesium) and 24-hour excretion of urine total protein and volume with **maximum ambient temperature,** adjusted for age, BMI, physical exercise, smoking, alcohol consumption, sleep duration, religion, household wealth, time of visit, drinking water salinity, and humidity.

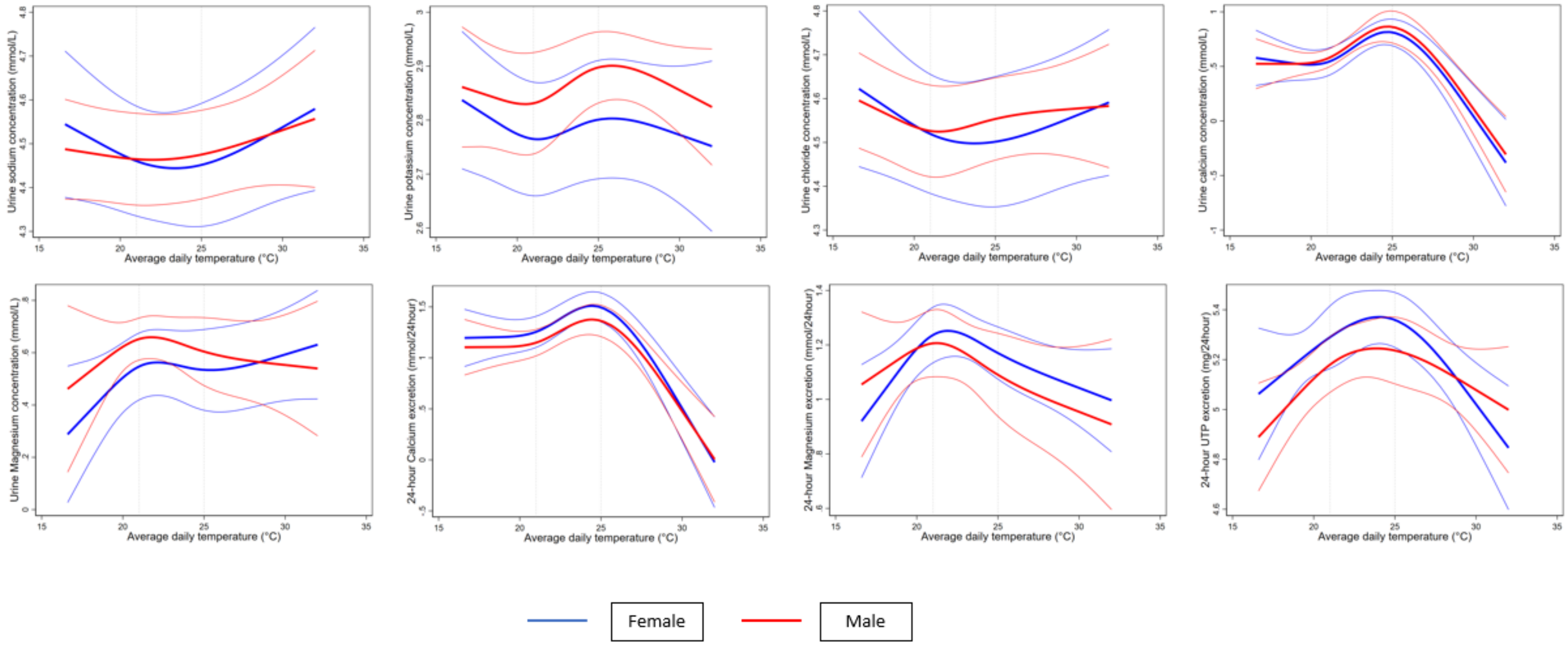


**Figure S12:** Restricted cubic spline plots with their 95% confidence bands using linear mixed-effect model demonstrating sex-stratified association between average temperature and urine electrolytes and total protein **after excluding outliers** with adjusting for age, BMI, physical exercise, smoking, alcohol consumption, sleep duration, religion, household wealth, time of visit, drinking water salinity, and humidity.

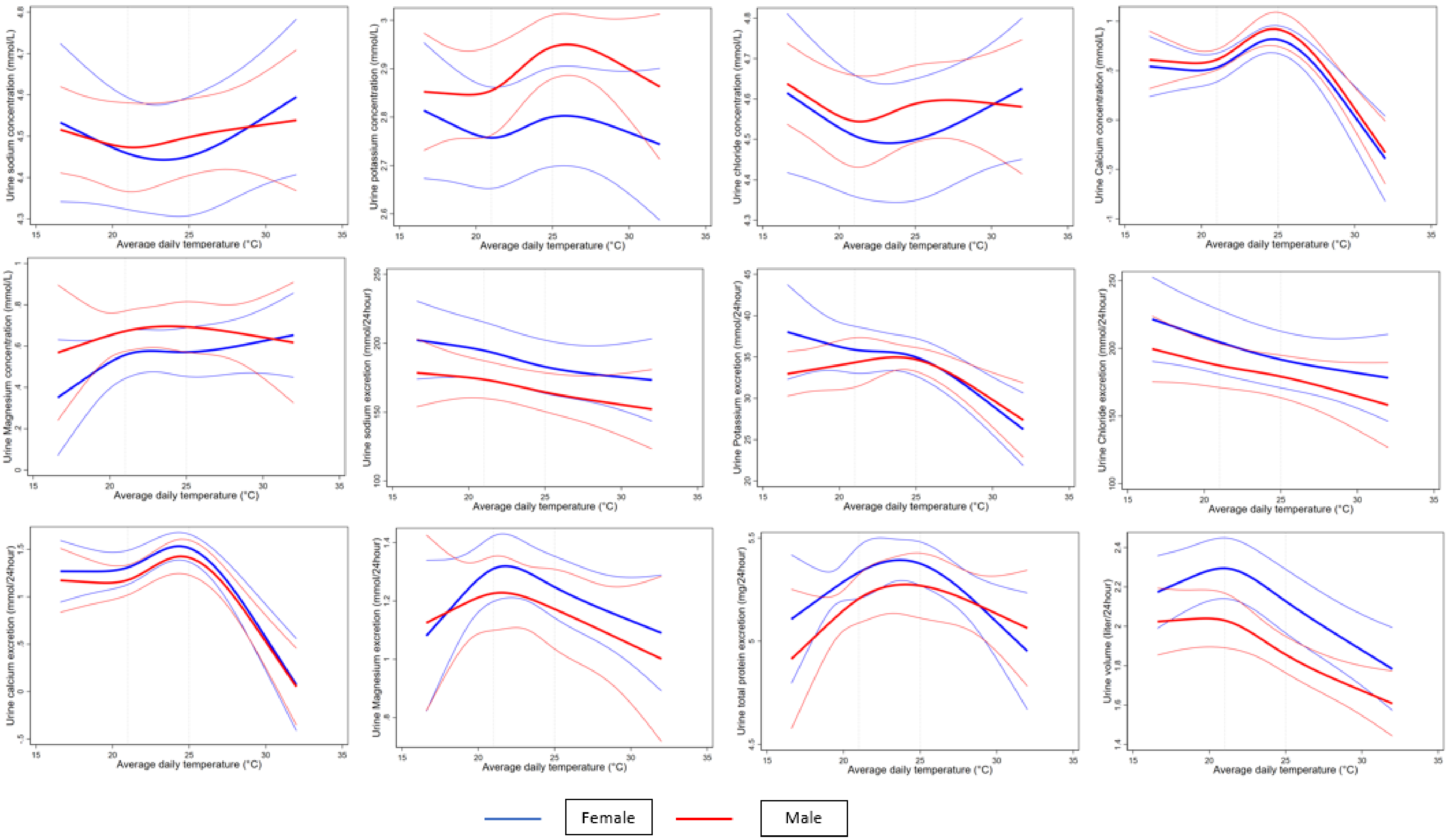


**Figure S13:** Restricted cubic spline plots with their 95% confidence bands using linear mixed-effect model demonstrating sex-stratified association of average ambient temperature with urine electrolytes, 24-hour urine total protein, and volume **among participants who did not report any comorbidities** after adjusting for age, BMI, physical exercise, smoking, alcohol consumption, sleep duration, religion, household wealth, time of visit, drinking water salinity, and humidity.

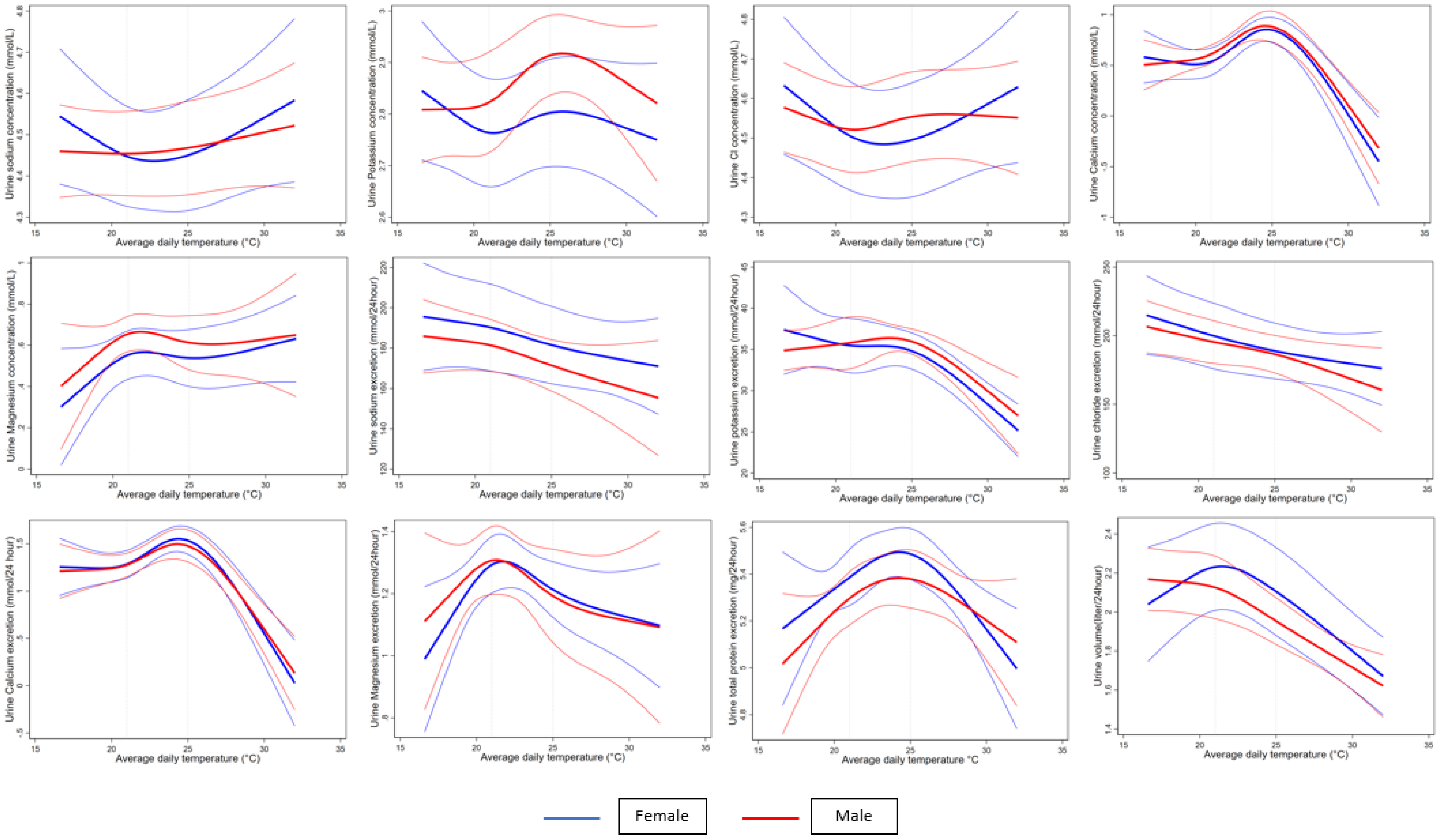


**Figure S14:** Restricted cubic spline (RCS) plots with their 95% confidence bands using a linear mixed-effect model demonstrating sex-stratified associations between the concentration and 24-hour excretion of urinary electrolytes (Sodium, Potassium, Chloride, Calcium, and Magnesium) and 24-hour urine total protein and volume with average ambient temperature **among participants with complete 24-hour urine collection based on creatinine index >0.7.** The RCS plots were adjusted for age, BMI, physical exercise, smoking, alcohol consumption, sleep duration, religion, household wealth, time of visit, drinking water salinity, and humidity.

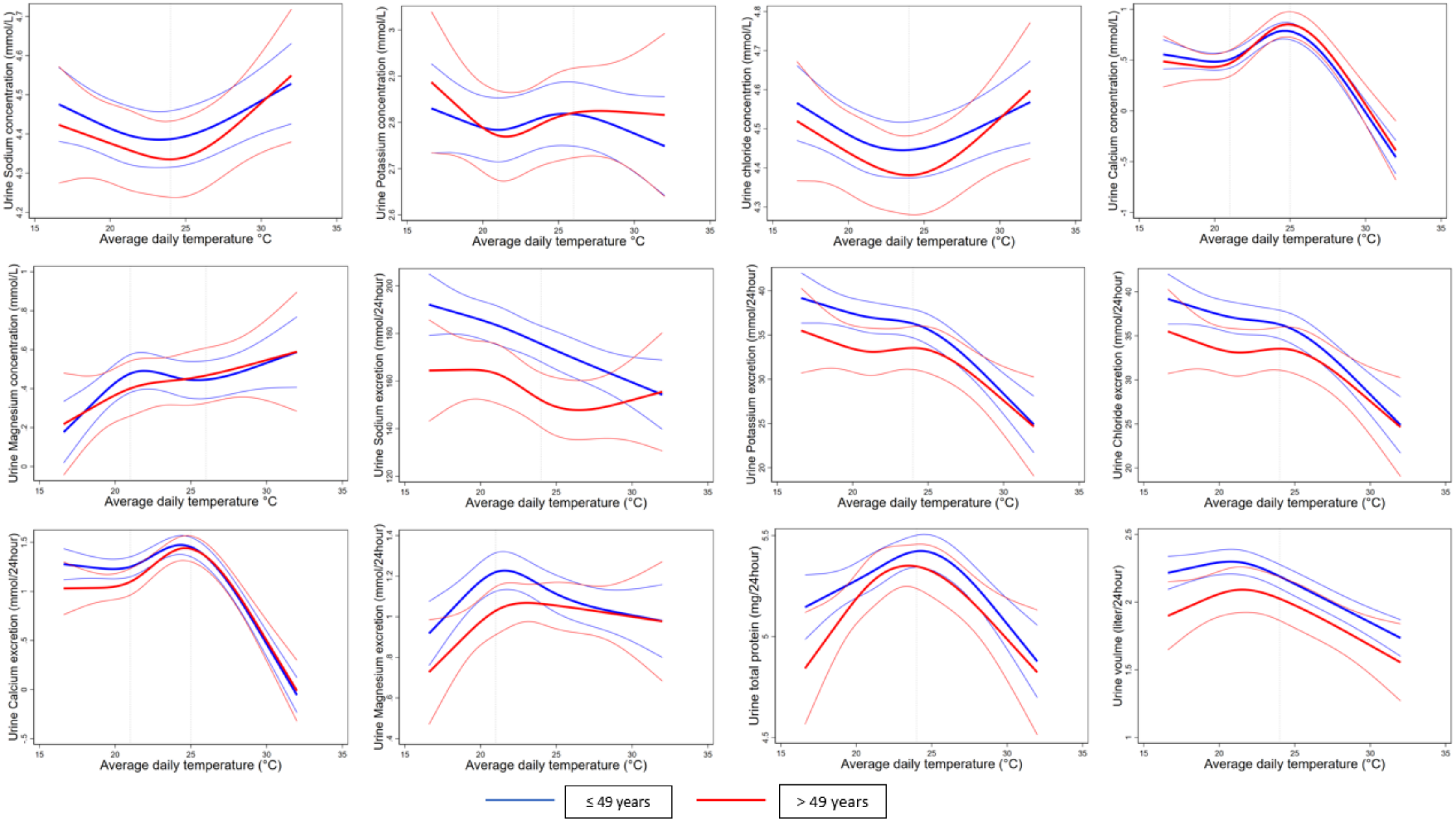


**Figure S15:** Restricted cubic spline plots with their 95% confidence bands using linear mixed-effect model demonstrating age-stratified association **amongst women** between the concentration and 24-hour excretion of urinary electrolytes (Sodium, Potassium, Chloride, Calcium, and Magnesium) and 24-hour excretion of urine total protein and urine volume with average ambient temperature, adjusted for other covariates.

STROBE Statement—checklist of items that should be included in reports of observational studies

| | Item No. | Recommendation | Page No. | Relevant text from manuscript |
|---|---|---|---|---|
| **Title and abstract** | 1 | (*a*) Indicate the study's design with a commonly used term in the title or the abstract | 3 | We evaluated the sex-specific association between ambient temperature and urinary biomarkers, including concentration and 24-hour excretion of electrolytes, and 24-hour total protein and volume, using longitudinal data of 1,175 participants' urine biomarkers (5624 person-visits) conducted in southwest coastal Bangladesh (Khulna, Satkhira, and Mongla districts) during November 2016 - April 2017 (Please refer to the ***Abstract***). |
| | | (*b*) Provide in the abstract an informative and balanced summary of what was done and what was found | 3 | Refer to the ***Abstract***. |
| **Introduction** | | | | |
| Background/ rationale | 2 | Explain the scientific background and rationale for the investigation being reported | 4-5 | Millions of people worldwide experienced higher ambient temperatures and were hurt by heatwaves during the summer of 2023. July 2023 was recorded as the world's hottest month ever since 1980, representing the fast-paced effect of climate change. Extreme heat exposure is linked to heat stress, heatstroke, kidney injury and exacerbation of congestive heart failure. Studies have identified a higher risk of nephropathies among outdoor agricultural workers attributed partly to higher ambient temperatures in Central American and South Asian countries. Epidemiological studies have reported that men are particularly at high risk of developing nephropathies and nephrolithiasis globally. Since men, as breadwinner, largely involve in outdoor work by exposing themselves directly under the sun, therefore prolong exposure to high ambient heat is very likely. However, women are also involved in strenuous outdoor activities in the vicinity of their homes and fields in many cultures, making them similarly vulnerable to ambient heat. Sex could be a biological variable that influences dimorphic physiological responses against ambient heat exposure, making men more vulnerable (Refer to the ***Introduction*** section). |

| | | | | |
|---|---|---|---|---|
| Objectives | 3 | State specific objectives, including any prespecified hypotheses | 5 | Unlike men, the health impact of chronic exposure to ambient heat on women is inconclusive, which demands much attention. Disaggregated data by sex and sex-stratified analyses considering the similar heat exposure across population may inform vulnerability and guide clinical and public health interventions to protect all groups affected from adverse health consequences of raised ambient temperature. In this study, we explored whether men and women have similar concentration and excretion of urinary electrolytes (sodium, potassium, calcium, magnesium, chloride), 24-hour excretion of urine total protein, and volume when exposed to a range of ambient temperatures spanning through the winter and summer months in a tropical coastal region of Bangladesh. (Refer to the ***last paragraph of the Introduction***). |
| **Methods** | | | | |
| Study design | 4 | Present key elements of study design early in the paper | 6 | We used urine electrolytes (sodium, potassium, chloride, calcium, and magnesium), total protein, and volume data from a stepped wedge randomized controlled trial conducted in three southwest coastal districts (Khulna, Satkhira, and Bagerhat) of Bangladesh from November 2016 to April 2017, covering both dry winter and early dry summer. The trial followed 1,175 participants from 542 households. We collected urine samples during five visits to evaluate the health effects of providing a low salinity drinking water intervention provided by managed aquifer recharge (MAR).<br>All participants gained access to the intervention water supply at some point in the study period during the second to fifth visits. Urine samples were collected from both intervention and control phases in all five consecutive months during the study period, making a total data of 5624 person visits. Detail of the study design, selection and enrolment of participants has been reported elsewhere. [22] (Refer to the ***"Study setting and design"*** part of ***Methods section***). |
| Setting | 5 | Describe the setting, locations, and relevant dates, including periods of recruitment, exposure, follow-up, and data collection | 6-8 | The detail of the study setting, locations, and relevant dates were described in "***Study setting and design"***, ***"Ambient temperature data"***, and ***"Urine sample collection and processing"*** part of the ***Methods section***. Data was collected from a stepped wedge randomized controlled trial conducted in 3 southwest coastal districts of Bangladesh. The detail of the study design, recruitment, follow-up, and data collection were published elsewhere.[22] |

| | | | | |
|---|---|---|---|---|
| Participants | 6 | (*a*) *Cohort study*—Give the eligibility criteria, and the sources and methods of selection of participants. Describe methods of follow-up<br>*Case-control study*—Give the eligibility criteria, and the sources and methods of case ascertainment and control selection. Give the rationale for the choice of cases and controls<br>*Cross-sectional study*—Give the eligibility criteria, and the sources and methods of selection of participants | 6 | This is a secondary analysis of 5624 person visits data collected from stepped wedge randomized controlled trial. Detail of the study design, selection and enrolment of participants has been reported elsewhere.[22] (Refer to ***"Study setting and design"*** part of the ***Methods section***). |
| | | (*b*) *Cohort study*—For matched studies, give matching criteria and number of exposed and unexposed<br>*Case-control study*—For matched studies, give matching criteria and the number of controls per case | | NA |
| Variables | 7 | Clearly define all outcomes, exposures, predictors, potential confounders, and effect modifiers. Give diagnostic criteria, if applicable | 6-8 | We have clearly defined and discussed all outcomes, exposure, and potential confounders/covariates in Method section (Refer to ***"Ambient temperature data"***, ***"Urine sample collection and processing"***, and ***"Confounders and covariates data"*** in the ***Methods section***). |
| Data sources/ measurement | 8* | For each variable of interest, give sources of data and details of methods of assessment (measurement). Describe comparability of assessment methods if there is more than one group | 7, 9-11 | We have discussed the details measurement process of each variable of interest in Method section (refer to ***"Urine sample collection and processing"*** and ***"Statistical analyses"*** in the ***Methods section***). |
| Bias | 9 | Describe any efforts to address potential sources of bias | 10-11 | Since we have 12 outcomes, we used Bonferroni correction to account for the multiple comparisons (e.g., urine volume and different biomarkers) in the final model (model 4) and we adjusted the alpha-level at 0.004 (0.05/12) for (α = |

| | | | | |
|---|---|---|---|---|
| | | | | 0.05/12 or 0.004) multiple comparisons. (Refer to the ***6th paragraph in “Statistical analyses”*** in the ***Methods section***). |
| Study size | 10 | Explain how the study size was arrived at | 6-7 | In this secondary analysis, we included entire 5624 person visits data from a stepped wedge cluster-randomized controlled trial and then linked with exposure variables (ambient temperature) by calculating shortest linear distances for each participant’s residence (Refer to ***“Study setting and design”*** and “***Ambient temperature data”*** in the ***Methods section***). The sample size calculation of original trial was described and published elsewhere[22] . |

Continued on next page

| | | | | |
|---|---|---|---|---|
| Quantitative variables | 11 | Explain how quantitative variables were handled in the analyses. If applicable, describe which groupings were chosen and why | 8 | We have given the information of quantitative variables in “Statistical analyses” (Refer to first paragraph in ***“Statistical analyses”*** part in the ***Methods section***). We also did provide the reason/explanation of grouping for some categorical variables in ***“Confounders and covariates data”*** part in Method section. The overview all variables were given in Table 1. |
| Statistical methods | 12 | (*a*) Describe all statistical methods, including those used to control for confounding | 9-11 | Refer to ***“Statistical analyses”*** part in the ***Methods section***. |
| | | (*b*) Describe any methods used to examine subgroups and interactions | 9-11 | This is a sex-stratified analysis to evaluate the association between ambient temperature and urine electrolytes and other urine markers. (Refer to ***“Statistical analyses”*** part in the ***Method section***). |
| | | (*c*) Explain how missing data were addressed | Not applicable | This is secondary analyses of the already collected data from a stepped wedge randomized controlled trial. We did complete case analysis of all available data. |
| | | (*d*) *Cohort study*—If applicable, explain how loss to follow-up was addressed<br>*Case-control study*—If applicable, explain how matching of cases and controls was addressed<br>*Cross-sectional study*—If applicable, describe analytical methods taking account of sampling strategy | Not applicable | |
| | | (*e*) Describe any sensitivity analyses | 10-11 | We conducted several sensitivity analyses. We did a stratified analyses for participants who did not self- report any co-morbidities (e.g., heart diseases, kidney disease, diabetes, and stroke). Since we collected 24-hour urine of the study participants in a rural non-clinical setting, there may be under-or over- collection of 24-hour urine samples. We used a creatinine-based measure of completeness assessment of 24-hour urine sample collection and implemented a stratified analyses among participants with complete 24-hour urine collection based on creatinine index > 0.7. Furthermore, stratified analyses were performed to create restricted cubic spline plots using linear mixed-effects models for women aged ≤ 49 years and ≥49 years. (Refer to the ***last paragraph of “Statistical analyses”*** in the ***Methods section***). |

| | | | | |
|---|---|---|---|---|
| **Results** | | | | |
| Participants | 13* | (a) Report numbers of individuals at each stage of study—eg numbers potentially eligible, examined for eligibility, confirmed eligible, included in the study, completing follow-up, and analysed | 12, Table 1 | This study involved urine biomarker data from 1,175 participants over 5,624 individual visits, derived from a stepped wedge randomized controlled trial. An overview of the characteristics of participants, including sex-stratified distribution, is presented in **Table 1**. (Refer to the ***Results section***). |
| | | (b) Give reasons for non-participation at each stage | NA | |
| | | (c) Consider use of a flow diagram | NA | |
| Descriptive data | 14* | (a) Give characteristics of study participants (eg demographic, clinical, social) and information on exposures and potential confounders | 12-13, Table 1, Figure 1 | The median age of the sample population was 41 years [Interquartile Range (IQR): 31-54]. The majority were women (59%), married (96%), and belonged to the Hindu religion (58 %). Fifty-one percent (51%) of study participants were never smokers, and 97% did not drink alcohol. An overview of the characteristics of participants, including sex-stratified distribution, is presented in **Table 1**. In the study area during the 5-month study period (December 2016-April 2017), the highest daily mean temperature was 30.4°C in April, while in January the lowest mean temperature was recorded (16.6 °C) (Refer to the ***1st paragraph of the Results, Table 1, and Figure 1***). |
| | | (b) Indicate number of participants with missing data for each variable of interest | Table 1 | This is a secondary analysis. An overview of each variable of interests is provided in ***Table 1***. |
| | | (c) *Cohort study*—Summarise follow-up time (eg, average and total amount) | Not available | |
| Outcome data | 15* | *Cohort study*—Report numbers of outcome events or summary measures over time | Not available | |
| | | *Case-control study*—Report numbers in each exposure category, or summary measures of exposure | Not available | |
| | | *Cross-sectional study*—Report numbers of outcome events or summary measures | 12-13, Table 2 & 3 | The findings of this secondary analyses are provided in the ***Results section***. The measures or estimates of all outcome variable following statistical analyses are provided in ***Table 2 & 3.*** |

| | | | | |
|---|---|---|---|---|
| Main results | 16 | (*a*) Give unadjusted estimates and, if applicable, confounder-adjusted estimates and their precision (eg, 95% confidence interval). Make clear which confounders were adjusted for and why they were included | Table 2,3; Table S1, S2, S3, S4; 4th paragraph of "Statistical analyses" in Method section (page 10) | The estimates of average ambient temperature and urine biomarkers in different models (model 1-4) are given in ***Table 2 and 3***. Furthermore, estimates of maximum and minimum ambient temperature and urine biomarkers in different statistical models (model 1-4) are reported in ***Table S1, S2, S3, and S4***. The detail description of different models is provided in the ***"Statistical analyses"*** in ***Methods section***. |
| | | (*b*) Report category boundaries when continuous variables were categorized | Not available | |
| | | (*c*) If relevant, consider translating estimates of relative risk into absolute risk for a meaningful time period | Not available | |

Continued on next page

| | | | | |
|---|---|---|---|---|
| Other analyses | 17 | Report other analyses done—eg analyses of subgroups and interactions, and sensitivity analyses | 12-13; Table 2,3 and Table S1, S2, S3, S4; Sensitivity analyses- Table S5 & S6. | The findings of this secondary analyses (sex-stratified mixed-effect linear regression) are described in the Result section. ***Table 2 and 3*** reported the associations between average ambient temperature and urine biomarkers, ***Table S1 and S2*** reported estimates about maximum ambient temperature and urine biomarkers, and ***Table S3 and Table S4*** reported estimates on the associations between minimum ambient temperature and urine biomarkers. Sensitivity analyses are reported in ***Table S5 and S6***. |
| **Discussion** | | | | |
| Key results | 18 | Summarise key results with reference to study objectives | 13 (1st paragraph in the Discussion section) | We found no sex difference in the statistical associations between ambient temperature and urine biomarkers, particularly sodium, chloride, potassium, calcium, magnesium, total protein, and urine volume. In contrast to many epidemiological studies highlighting men particularly vulnerable to high ambient heat, our results suggest women are equally susceptible to ambient temperature-related urinary biomarkers changes. We found higher urine sodium and chloride concentrations but lower 24-hour excretions for both sexes when ambient temperature increased, this is consistent with prior evidence and suggest substantial sweating by both men and women (Refer to the ***1st paragraph in Discussion section***). |
| Limitations | 19 | Discuss limitations of the study, taking into account sources of potential bias or imprecision. Discuss both direction and magnitude of any potential bias | 15-16 (7th and 8th paragraph in Discussion) | We have discussed the plausible limitations of this study including its potential impact (Refer to the ***7th and 8th paragraph in Discussion section***). |
| Interpretation | 20 | Give a cautious overall interpretation of results considering objectives, limitations, multiplicity of analyses, results from similar studies, and other relevant evidence | 13-16 | We have provided the overall interpretation of the results with supporting evidence, limitations, results from similar studies and other relevant evidence in the ***Discussion section***. |
| Generalisability | 21 | Discuss the generalisability (external validity) of the study results | 16 | We believe this study findings are generalizable to the population with similar coastal and climatic contexts, including saltwater intrusion affected areas where communities experience drinking water salinity (e.g., Ganges River delta, Mekong, and Red River delta).<br>(Refer to ***last paragraph in the discussion***). |

| | | | | |
|---|---|---|---|---|
| **Other information** | | | | |
| Funding | 22 | Give the source of funding and the role of the funders for the present study and, if applicable, for the original study on which the present article is based | 25 | The source of funding of the original randomized controlled trial is provided in ***page 18***. |

*Give information separately for cases and controls in case-control studies and, if applicable, for exposed and unexposed groups in cohort and cross-sectional studies.

**Note:** An Explanation and Elaboration article discusses each checklist item and gives methodological background and published examples of transparent reporting. The STROBE checklist is best used in conjunction with this article (freely available on the Web sites of PLoS Medicine at http://www.plosmedicine.org/, Annals of Internal Medicine at http://www.annals.org/, and Epidemiology at http://www.epidem.com/). Information on the STROBE Initiative is available at www.strobe-statement.org.

**Reference list:**

22. Naser AM, Unicomb L, Doza S, et al. Stepped-wedge cluster-randomised controlled trial to assess the cardiovascular health effects of a managed aquifer recharge initiative to reduce drinking water salinity in southwest coastal Bangladesh: study design and rationale. *BMJ Open*. 2017;7(9):e015205. doi:10.1136/bmjopen-2016-015205
